# NSUN2 lactylation drives cancer cell resistance to ferroptosis through enhancing GCLC-dependent glutathione synthesis

**DOI:** 10.1016/j.redox.2024.103479

**Published:** 2024-12-19

**Authors:** Kaifeng Niu, Zixiang Chen, Mengge Li, Guannan Ma, Yuchun Deng, Ji Zhang, Di Wei, Jiaqi Wang, Yongliang Zhao

**Affiliations:** a China National Center for Bioinformation, Beijing, 100101, China; b Beijing Institute of Genomics, Chinese Academy of Sciences, Beijing, 100101, China; c University of Chinese Academy of Sciences, Beijing, 100049, China; d Key Laboratory of Digital Technology in Medical Diagnostics of Zhejiang Province, Hangzhou, 310030, China

**Keywords:** NSUN2, Lactylation, RNA 5-methylcytosine, GCLC, Glutathione synthesis, Ferroptosis

## Abstract

Lactate-mediated lactylation on target proteins is recently identified as the novel posttranslational modification with profound biological functions. RNA 5-methylcytosine (m^5^C) modification possesses dynamic and reversible nature, suggesting that activity of its methyltransferase NSUN2 is actively regulated. However, how NSUN2 activity is response to acidic condition in tumor microenvironment and then regulates cancer cell survival remain to be clarified. Here, we demonstrate that NSUN2 activity is enhanced by lactate-mediated lactylation at lysine 508, which then targets glutamate-cysteine ligase catalytic subunit (GCLC) mRNA to facilitates GCLC m^5^C formation and mRNA stabilization. The activated GCLC induces higher level of intracellular GSH accompanied by decreased lipid peroxidation and resistant phenotype to ferroptosis induction by doxorubicin (Dox) in gastric cancer cells. Specifically, the effect of NSUN2 lactylation-GCLC-GSH pathway is nearly lost when NSUN2 K508R or GCLC C-A mutant (five cytosine sites) was introduced into the cancer cells. We further identify the catalytic subunit N-α-acetyltransferase 10 (NAA10) as the lactytransferase of NSUN2, and lactate treatment substantially enhances their association and consequent NSUN2 activation. Taken together, our findings convincingly elucidate the signaling axis of NAA10-NSUN2-GCLC that potently antagonizes the ferroptosis under acidic condition, and therefore, targeting NSUN2 lactylation might be an effective strategy in improving the prognosis of cancer patients.

## Highlights

• Lactate-mediated NSUN2 lactylation promotes its catalyzing activity.
• NSUN2 lactylation promotes GSH synthesis through stabilizing GCLC mRNA.
• NAA10 catalyzes lactylation of NSUN2 rendering cancer cell ferroptosis resistant.

## Graphical abstract

**Figure:**
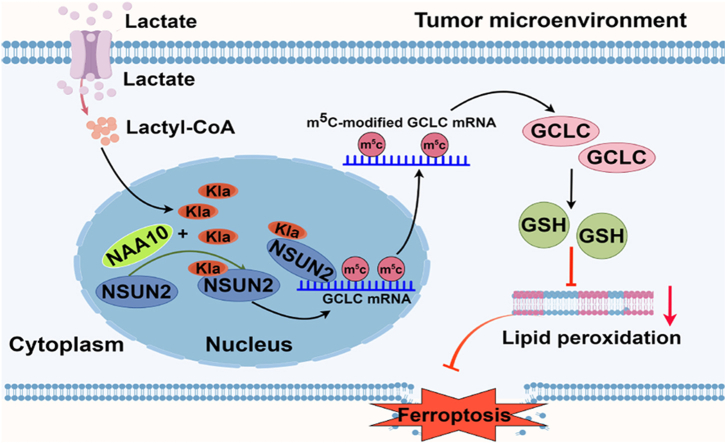

## 1 Introduction

Acidic tumor microenvironment (TME) is the characteristic feature of tumor cells due to enhanced aerobic glycolysis that releases excessive amount of lactate into the extracellular environment, which consequently promotes tumor growth, angiogenesis, metastasis, drug resistance, and immunosuppression [1]. Blockage of either lactate production or transport has become a promising target in cancer therapy [2,3]. Since lactate-mediated lysine lactylation (Kla) modification on histone protein has first been discovered in 2019, lactylation on non-histone proteins were also demonstrated [4,5]. Further evidences confirm that lactylation serves as a new type of epigenetic modification and a vital component of lactate function implicated in various biological processes, such as homologous recombination (HR)-mediated DNA repair [6,7], cell fate determination [8], neurodegenerative disease [9], tumor progression [10,11] and drug resistance [6,12].

Just like the potential effectiveness of lactate blockage in cancer treatment, suppression of lactate-derived lactylation may also represent a promising target with therapeutic value. Yang et al. used mass spectrum analysis to screen for differentially lactylated proteins between clinical intrahepatic cholangiocarcinoma (iCCA) and paracancerous tissues, and found that nucleolin (NCL) is lactylated at lysine 477 in response to a hyperactivity of glycolysis and potentiates iCCA pathogenesis via the MAPK pathway [13]. Histone lactylation (H3K18la) can activate mitotic checkpoint regulator TTK protein kinase (TTK) to promote the progression of pancreatic ductal adenocarcinoma (PDAC) [14], while suppression of H3K18la by circXRN2 (hsa_circ_0001134) inhibits tumor progression through activating the Hippo signaling pathway in human bladder cancer [15]. In addition, stiripentol, a lactate dehydrogenase (LDH) A inhibitor, was shown to inhibit NBS1 K388 lactylation to decrease DNA repair efficacy and sensitize phenotype to chemotherapy [6]. Downregulation of MRE11 lactylation by inhibition of CREB binding protein (CBP) or LDH impaired the homologous recombination repair leading to enhanced chemosensitivity in patient-derived xenograft and organoid tumor models [7]. These reports suggest important roles of lactate-mediated lactylation in regulating tumorigenenic process.

NSUN2 is a nuclear methyltransferase and responsible for the m^5^C modification in RNA [16]. Its aberrant overexpression has been found in multiple types of human cancers and associated with various biological processes, such as cell differentiation and early embryogenesis, cell proliferation, migration and tumor progression [[17], [18], [19], [20]]. In particular, NSUN2 can increase gene expression by targeting the RNA metabolism, such as stability, nuclear export and translation [16]. Silence of NSUN2 led to decreased m^5^C modification level concomitant with reduced mRNA expression of many genes (QSOX1, FABP5, IRF3, SLC7A11, IL17a and IL17f) that regulate drug resistance [18], fatty acid metabolism [21], ferroptosis [22], and inflammatory response [23,24]. Interestingly, NSUN2, as a direct glucose sensor that can be activated by glucose, drives tumorigenesis and immunotherapy resistance by TREX2-mediated cGAS/STING inactivation [25]. However, in addition to the above transcriptional regulation, it is still largely unknown about the posttranslational modifications (PTMs) and functional regulation for NSUN2. One recent report shows that a small ubiquitin-like modifier (SUMO)-2/3 directly interacts and stabilizes NSUN2 which is implicated in tumor progression [26].Lactic acid was observed to activate the transcription of NSUN2 through histone H3K18 lactylation (H3K18la), and also induce the lactylation of NSUN2 [27], however, whether NSUN2 lactylation regulates its methyltransferase activity and further biological function remains to be clarified.

Acidic condition has been associated with an increased cellular level of reactive oxygen species [28,29], which can induce regulated cell death (RCD), such as apoptosis and ferroptosis [3,30]. Ferroptosis is an identified form of RCD driven by iron-dependent phospholipid (PL) peroxidation [31], and resistance to RCDs is the typical phenotype of cancer cells during tumor progression [32]. Moreover, cancer cells present highly heterogeneous metabolic profiles in individual tumor resulting from diverse cues in the tumor microenvironment and genetic mutations [33]. Lactic acid is one of the most abundant metabolites in the tumor microenvironment [34], and can mediate lysine lactylation on histone and non-histone proteins which have been shown to regulate metabolic pathways of cancer cells, such as glycolysis, fatty acid synthase and carbohydrate metabolism [10,35,36]. Thinking of the dynamic and reversible nature of RNA methylation [37], it is likely that NSUN2 activity is actively regulated by lactate-mediated lactylation, which then modulates the m^5^C level and stability of target genes eventually facilitating cancer cell survival under acidic TME condition. To prove this hypothesis, we validated the existence of lactylation modification on NSUN2, and determined how this modification affects its catalyzing activity. We further demonstrated that GCLC mRNA is a target of lactylated NSUN2, which coordinately regulates the lipid peroxidation level and the phenotype to ferroptosis resistance in gastric cancer cells. In addition, NAA10 was identified as the lactytransferase of NSUN2, and observed an enhanced association between these two proteins under lactate treatment. Overall, our present study demonstrated that NAA10-catalyzed lactylation of NSUN2 potently promotes cancer cell survival through a GCLC-dependent glutathione synthesis.

## 2 Methods

*Cell culture and Chemicals***:** Cell lines (HEK293, SNU-1, SNU-16, AGS) were obtained from the American Type Culture Collection, MKN45 and HGC-27 were ordered from Japanese Collection of Research Bioresources and the European Collection of Cell Culture. BGC823 were ordered from Hunan Fenghui Biotechnology Co., Ltd, China. All cell lines were cultured in DMEM (Gibco) containing 10 % fetal bovine serum (Gibco). Mycoplasma was tested as negative using Mycoplasma Detection Kit (C0301S, Beyotime).

The following chemical reagents were used: RSL3 (SML2234, Sigma-Aldrich), Ferrostatin 1 (ab146169, Abcam), Z-VAD-FMK (S7023, Selleck), C11 BODIPY 581/591 (27,086, Cayman), Doxorubicin (E2516, Selleck), Sodium oxamate (ab145643, abcam), L-(+)-Lactic acid (L6402, Sigma Aldrich) and Rotenone (R8875, Sigma Aldrich).

*Western blotting and antibodies***:** Proteins were analyzed by western blotting according to the standard methods. The antibodies that were used are as follows: FLAG (F3165) and GAPDH (MAB374) from Sigma-Aldrich; HA (51064-AP), NSUN2 (20854-1-AP), NAA10 (14803-1-AP) and m^5^C (68301-1-Ig) from Proteintech; pan-acetyllysine (PTM-101) and pan-l-Lactyllysine (PTM-1401RM) from PTM BIO; GCLC (ab190685, Abcam), SLC7A11 (A2413, Abclonal), GCLM (AF6972, Beyotime).

*RNA interference***:** For knockdown assay, control siRNA or siRNAs targeting NSUN2 or NAA10 were transfected into MKN45 cells. Rfect siRNA transfection reagent (110,113, Baidai biotechnology) was used. The following siRNA oligo sequences were used:

*NSUN2* siRNA1: 5′-GAGAUCCUCUUCUAUGAUCGATT-3’;

*NSUN2* siRNA2: 5′-CACGUGUUCACUAAACCCUAUTT-3’;

*NAA10* siRNA1: 5′-GCCCAAAUACUAUGCAGAUTT-3’;

*NAA10* siRNA2: 5′-GGCCAGAGGACCUAAUGAATT-3’;

Control siRNA: 5′-UUCUCCGAACGUGUCACGUTT-3’.

*Generation of NSUN2 knockout MKN45 cells***:** The NSUN2 knockout (KO) MKN45 cell line was generated by CRISPR-Cas9 system. Two guide RNA (sgRNA) sequences (5′-GTGTTCACTAAACCCTATTG-3’; 5′-GGATGCCTGGAATCACACAG-3′) were designed and the sgRNA oligonucleotides were synthesized and inserted into PX330-GFP-U6 vector. The above plasmids were transfected into MKN45 cells for 48 h followed by flow cytometry sorting to gain targeted cell clones. Genotype and gene silencing efficiency of cells were assessed using PCR and Western blotting.

*Co-immunoprecipitation (co-IP) assay and LC-MS/MS analysis*: HEK293 cells transiently transfected with p3xFlag-NSUN2 plasmid were lysed by the IP buffer containing 150 mM NaCl, 50 mM Tris-HCl, pH 7.5, 1 % NP-40, 5 mM EDTA and protease inhibitor cocktail (B14001, Bimake) at 4 °C. After centrifugation, the Flag-M2 beads (B23102, Bimake) and supernatant were co-incubated for 2 h at 4 °C. After washed with IP buffer for three times, the beads were finally boiled at 95 °C in 2 × SDS sample buffer followed by silver staining and LC-MS/MS analysis.

*Untargeted metabolomics methods and analysis:* Untargeted metabolomics analysis was performed by Shanghai Applied Protein Technology Co., Ltd. In brief, NSUN2 WT and KO MKN45 cells (∼10^7^ cells per sample) with each containing 6 technical replicates were washed twice with cold PBS and then once with cold normal saline solution (0.9 % NaCl). The samples were treated with 1 ml of cold methanol/acetonitrile/H2O (2:2:1, v/v/v) to remove the protein for metabolite extraction. The mixture was centrifuged at 14,000 g for 20 min at 4 °C, and the supernatant was collected and dried in a vacuum centrifuge. For LC-MS analysis, the samples were re-dissolved in 100 μl acetonitrile/water (1:1, v/v) solvent, and analyzed using a UHPLC system (1290 Infinity LC, Agilent Technologies) coupled to a quadrupole time-of-flight (AB Sciex TripleTOF 6600). The stability and repeatability of the instrument analysis were monitored using quality control (QC) samples.

For data analysis, the raw MS data were converted to MzXML files using ProteoWizard MSConvert before importing into freely available XCMS software. Following peak picking and peak grouping, CAMERA (Collection of Algorithms of MEtabolite pRofile Annotation) was used for annotation of isotopes and adducts. In the extracted ion features, only the variables having more than 50 % of the nonzero measurement values in at least one group were kept. Compound identification of metabolites was performed by comparing of accuracy and MS/MS spectra with an in-house database established with available authentic standards. Student's t-test was used to determine the significance of differences between two groups of independent samples. VIP >1 and *p* value < 0.05 were used to screen significant changed metabolites.

Metabolite set enrichment analysis (MSEA) was performed using MetaboAnalyst 6.0 software (https://www.metaboanalyst.ca/MetaboAnalyst/home.xhtml) [38]. The reference library of the Human Metabolome Database (HMDB) was used to identify the metabolite-enriched pathways. *P* values were adjusted using the Holm-Bonferroni method (Holm *P*).

*Cysteine assay:* Cysteine concentration was measured by Cysteine assay kit (KTB1450, Abbkine) according to the manufacturer's instructions. In brief, NSUN2 WT or KO MKN45 cells (5 × 10^6^ cells per sample) were washed twice with cold PBS and centrifuged at 1000 rpm for 5 min. The cell pellet was suspended in 1 ml Extraction Buffer and subjected to ultrasonic disruption (power 200 W, ultrasonic 3 s, interval 7 s, repeat 30 times). After centrifugation at 13,000 rpm for 10 min at 4 °C, the Cysteine content in the supernatant was calculated by measuring A600 according to the manufacturer's instructions.

*Glutamate assay:* Glutamate concentration was measured by Glutamate assay kit (MAK004, Sigma-Aldrich) according to the manufacturer's instructions. In brief, NSUN2 WT or KO MKN45 cells (1 × 10^6^ cells per sample) were washed twice with cold PBS and centrifuged at 1000 g for 5 min. The cell pellet was homogenized using 100 μl of the Glutamate Assay Buffer and then centrifuged at 13,000 g for 10 min at 4 °C. The glutamate content was calculated by measuring A450 according to the manufacturer's instructions

*m*^*5*^*C dot blot assay*: Total RNAs were isolated using the Trizol reagent (15596018, Invitrogen) and denatured at 70 °C for 2 min, followed by chilling on ice directly. The sample (500 ng −1000ng) were deposited onto a Hybond-N+ membrane (RPN303B, GE Healthcare) and air-dried for 5 min. The membrane was cross-linked by 254 nm-UV light for 3 min and blocked with 5 % nonfat milk in TBST for 1 h, followed by incubation with anti-m5C antibody (68301-1-Ig, Proteintech) overnight at 4 °C. The secondary antibody (PI-2000-1, Vector) was incubated with the membrane for 1 h at room temperature. The spots were visualized using an imaging system. Staining with 0.02 % with methylene blue (M9140, Sigma Aldrich) was used to verify an equal amount of RNA spotted on the membrane.

*RNA extraction and quantitative real-time polymerase reaction (qRT-PCR)*: The total RNA was extracted from HEK293 or MKN45 cells using the Trizol reagent and single-strand cDNA was reversely transcribed with RevertAid First Strand cDNA Synthesis Kit (K1622, Thermo Scientific). Real-time PCR was performed using SYBR Premix Ex Taq (RR420A, TaKaRa) and the plates were read with Bio-Rad CFX96. The Primers used for qPCR analysis were listed below:

*GCLC*-F: 5′-AAAAGTCCGGTTGGTCCTG-3’

*GCLC*-R: 5′-CCTGGTGTCCCTTCAATCATG-3’

*GCLM*-F: 5′-ACTAGAAGTGCAGTTGACATGG-3’

*GCLM*-R: 5′-AGGCTGTAAATGCTCCAAGG-3’

*GAPDH*-F: 5′-GGAGCGAGATCCCTCCAAAAT-3’

*GAPDH*-R: 5′-GGCTGTTGTCATACTTCTCATGG-3’

*RNA immunoprecipitation (RIP) assay*: The binding between NSUN2 protein and GCLC mRNA was assessed using the RNA Immunoprecipitation (RIP) Kit (Bes5101, BersinBio). Protein A/G magnetic beads were pre-washed and incubated with 5 μg of anti-NSUN2 antibody at 4 °C overnight and subjected to immunoprecipitation. The RNA was extracted and purified using the phenol-chloroformethanol method. Enrichment was calculated by qPCR and the corresponding GCLC mRNA enrichment in each sample was calculated by normalizing to the input.

*Methylated RNA immunopreciptitation (MeRIP)*: The m5C modifications on GCLC mRNA were determined using the MeRIP kit (Bes5204, BersinBio). Briefly, about 100 μg total RNA was isolated using Trizol reagent and fragmented using RNA fragmentation buffer at 94 °C for 2 min. One-tenth of the fragmented RNA was preserved as the input group, and other cleaved RNA fragments were incubated with 4 μg m^5^C antibody at 4 °C for 4 h with rotation. Subsequently, 20 μL A/G magnetic beads were prewashed and added into the above mixture at 4 °C for 1 h followed by washing three times. The bound RNA fragments were eluted from the beads by proteinase K digestion at 55 °C for 30 min. The RNAs were isolated from the eluate by phenol-chloroform extraction for subsequent qRT-PCR analysis.

*Dual-luciferase reporter assay*: Luciferase reporter plasmids containing CMV promoter and CDS-GCLC-wild type or GCLC-mutants (5-UTR mutation:C13A; CDS mutations:C81A, C972A, C1135A, C1136A) were synthesized by Sangon Biotech (Shanghai) Co., Ltd and cloned into pGL6-TA vector (D2105, Beyotime). The luciferase reporter plasmids and Renilla luciferase control reporter vectors were transfected into NSUN2 KO cells using lipomax (LipoMax32012, Sudgen). After 48 h, these cells were lysed and subjected to the luciferase activity analysis using Dual-Glo® Luciferase Assay System (E2920, Promega).

*RNA stability assay*: NSUN2 wild type and KO MKN45 cells were seeded in 6 cm dish and treated with Actinomycin D (5 μg/mL, S8964, Selleck) for 0, 3 and 6 h. Total RNA was extracted by TRIzol reagent, and equal amount of RNA for each treatment was used for qRT-PCR to detect the mRNA level of GCLC. The mRNA half-life time was calculated according to the linear regression analysis [72].

*Lactate assay*: l-Lactate concentration was measured by l-lactate assay kit (BC2235, Solarbio) according to the manufacturer's instructions. In brief, HEK293 cells were transfected with Flag-NSUN2 for 48 h followed by the treatment of DMSO or 50 μM rotenone for 24 h. The treated cells (1 × 10^6^ cells per sample) were washed twice with cold PBS and centrifugated at 1000 rpm for 5 min. The cell pellet was suspended in 1 ml Extraction Buffer I, and subjected to ultrasonic disruption (power 300 W, ultrasonic 3 s, interval 7 s, repeat 30 times). After centrifugation (13,000 rpm, 10 min at 4 °C), the supernatant (about 0.8 ml) was collected and mixed with 0.15 ml Extraction Buffer II. After centrifugation (13,000 rpm, 10 min at 4 °C), the lactate content was calculated by measuring A570 according to the manufacturer's instructions.

*Cell viability assay*: The cellular viability was measured by a Cell Counting Kit 8 (CCK8, C0042, Beyotime). In brief, the cell suspension (5000 cells per well) was seeded in a 96-well plates and incubated in a CO_2_ incubator (5 % CO_2_) at 37 °C for 24 h. 10 μL CCK-8 solution was added into each of the above wells and cells were incubated again for 1 h. Absorbance was measured at 450 nM wavelength. In total ≥ six control wells were included for each time point in all experiments.

*Glutathione (GSH) assay*: The total GSH level was measured using Total Glutathione Assay Kit (S0052, Beyotime). In brief, NSUN2 WT or KO MKN45 cells (1 × 10^6^ cells per sample) were washed twice with cold PBS and centrifugated (1000 rpm, 5 min). The cell pellet was suspended in 100 μl protein removal reagent S solution and then freeze-thawed twice rapidly using liquid nitrogen and 37 °C water bath. The samples were placed on ice for 5 min and centrifugated at 13,000 rpm for 20 min at 4 °C. The total glutathione content in the supernatant was calculated by measuring A412 according to the manufacturer's instructions.

*Lipid peroxidation analysis using C11-BODIPY*: Lipid peroxidation level was detected by C11-BODIPY 581/591 (D3861, Invitrogen). MKN45 cells (5000–10,000 cells per well) were seeded in 12-well plates and treated with Doxorubicin. 2 μM C11-BODIPY 581/591 was added and incubated for at 37 °C for 30 min. These cells were harvested and resuspended in PBS plus 5 % FBS followed by flow cytometry.

*Statistical analysis*: All experimental data were presented as the mean ± standard deviation (SD) of at least three independent experiments. Two-way ANOVA or paired Student's *t*-test were performed to determine significance using GraphPad Prism (GraphPad Prism version 9.0 for Windows) followed by Tukey post hoc test. ∗*P* < 0.05, ∗∗*P* < 0.01 or ∗∗∗*P* < 0.001 indicated that the difference was statistically significant.

## 3 Results

### 3.1 NSUN2 undergoes lactylation modification under lactate treatment

Methyltransferase-NSUN2 triggers m^5^C formation in mRNA and tRNA with profound biological functions [16,25]. To determine whether lactate regulates NSUN2 activity through mediating its lactylation, we first analyzed the global m^5^C methylation level in the presence or absence of lactate treatment using a dot blot assay. As glucose can be directedly metabolized into lactate [39], we chose the condition of glucose starvation plus lactate treatment. HEK293T cells transfected with 3 × Flag-NSUN2 plasmids were glucose-starved for 3 h followed by the treatment of 10 or 20 mM lactate acid for 6 h. The result showed that glucose deprivation significantly decreases m^5^C methylation level, while supplement of lactate significantly reverses this trend (Fig. 1A), indicating that the cellular m^5^C methylation level is affected by lactate. We then employed the co-immunoprecipitation (co-IP) assay to examine the lactylation status of NSUN2, and the result displayed a substantially enhanced lactylation level under different concentrations of lactate (10 and 20 mM) (Fig. 1B) or different treatment times (3 and 6 h) at 20 mM (Fig. 1C). In addition, endogenous NSUN2 was also observed to be lactylated with an enhanced level under lactate treatment in MKN45 gastric cancer cells (Fig. 1D). Inhibition of lactate production by using lactate dehydrogenase inhibitor-oxamate [4] in Flag-NSUN2 overexpressing HEK293 cells markedly decreased NSUN2 lactylation level (Fig. 1E). In contrast, treatment with rotenone that inhibits electron transfer from complex I to ubiquinone and drives cells towards glycolysis [40] induced a significant increase in intracellular lactate level and a substantial enhancement of NSUN2 lactylation in HEK293 cells (Fig. 1F and G). These data prove that lactate directly mediates the lactylation of NSUN2.

**Fig. 1:**
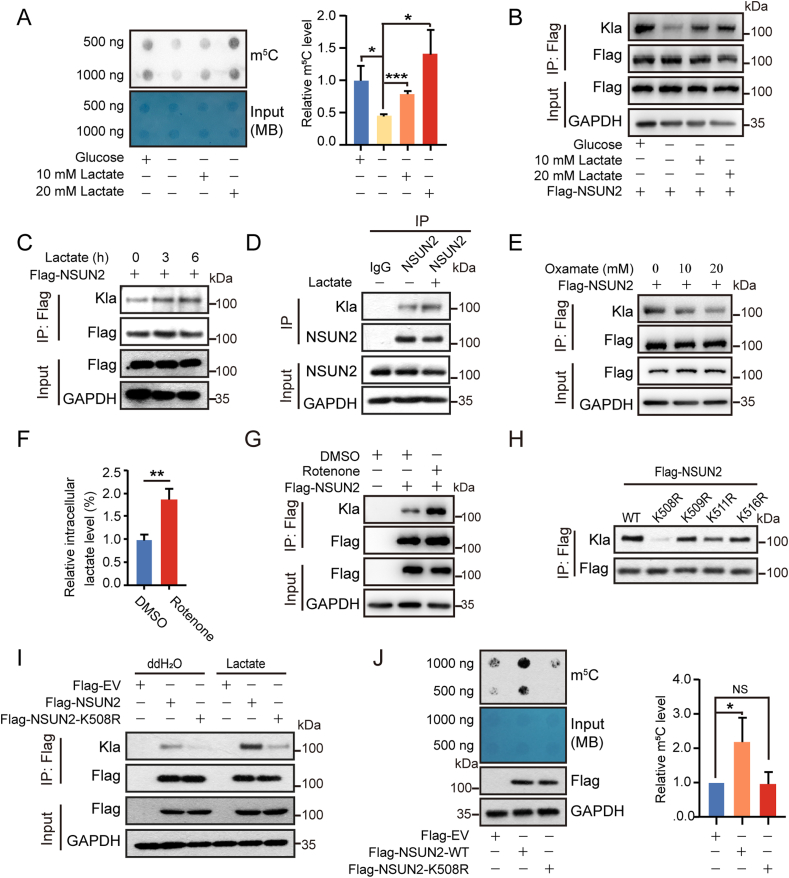
NSUN2 is activated and lactylated at K508 by lactate. (A, B) HEK293 cells were transfected with Flag-NSUN2 for 48 h, followed by glucose starvation for 3 h and treatment with or without 10 or 20 mM lactate for 6 h. (A) Dot blot assay measured total RNA m^5^C levels and its intensity of (A) was calculated using the Image J. (B) NSUN2 lactylation level in Flag immunoprecipitates was examined by western blotting. (C) HEK293 cells transfected with Flag-NSUN2 were treated with 20 mM lactate for 3 or 6 h. The lactylation level of NSUN2 was analyzed by co-IP and western blotting. (D) MKN45 cells were treated with or without 20 mM lactate for 24 h, and NSUN2 lactylation level was detected with anti-lactyllysine antibody. (E–G) HEK293 cells were transfected with Flag-NSUN2 for 48 h followed by the treatment of oxamate (E) or rotenone (F and G). The NSUN2 lactylation was examined via western blotting (E and G). Intracellular lactate levels were measured in HEK293 cells (F). (H) HEK293 cells were transfected with Flag-NSUN2-WT or Flag-NSUN2-mutations (K508R, K509R, K511R or K516R) for 48 h. The NSUN2 lactylation level was detected in anti-Flag immunoprecipitates and western blotting. (I) HEK293 cells transfected with Flag-NSUN2-WT or Flag-NSUN2-K508R were treated with or without 20 mM lactate for 24 h. The lactylation level of NSUN2 was analyzed by co-IP and western blotting. (J) Flag-NSUN2-WT or Flag-NSUN2-K508R were transfected into HEK293 cells for 48 h. Dot blot assay measured total RNA m^5^C levels and its intensity of (J) was calculated using the Image J. The data represent the mean ± SD from three independent experiments. Statistical analysis by paired Student's *t*-test. ∗P < 0.05, ∗∗P < 0.01, ∗∗∗P < 0.001, NS: not significant.

To search for the lysine sites undergoing lactylation on NSUN2, all the 57 lysine residues was screened by a point-mutation assay (lysine mutated to arginine), followed by co-IP and western blotting analyses. As shown in Fig. 1H and Fig. S1, NSUN2–K508R had a markedly reduced lactylation level compared to that of wild type (WT) NSUN2, and the effect was more pronounced upon lactate treatment (Fig. 1I), suggesting that K508 is the main site that undergoes lactylation. Interestingly, overexpression of WT NSUN2, but not K508R mutant, in HEK293 cells markedly increased the global m^5^C RNA methylation level (Fig. 1J), pointing to the critical role of NSUN2 lactylation in enhancing its activity and subsequent m^5^C modification level in its mRNA targets.

### 3.2 Lactylation of NSUN2 enhances its catalyzing activity and stabilizes GCLC mRNA via promoting m^5^C modification

**Fig. 2:**
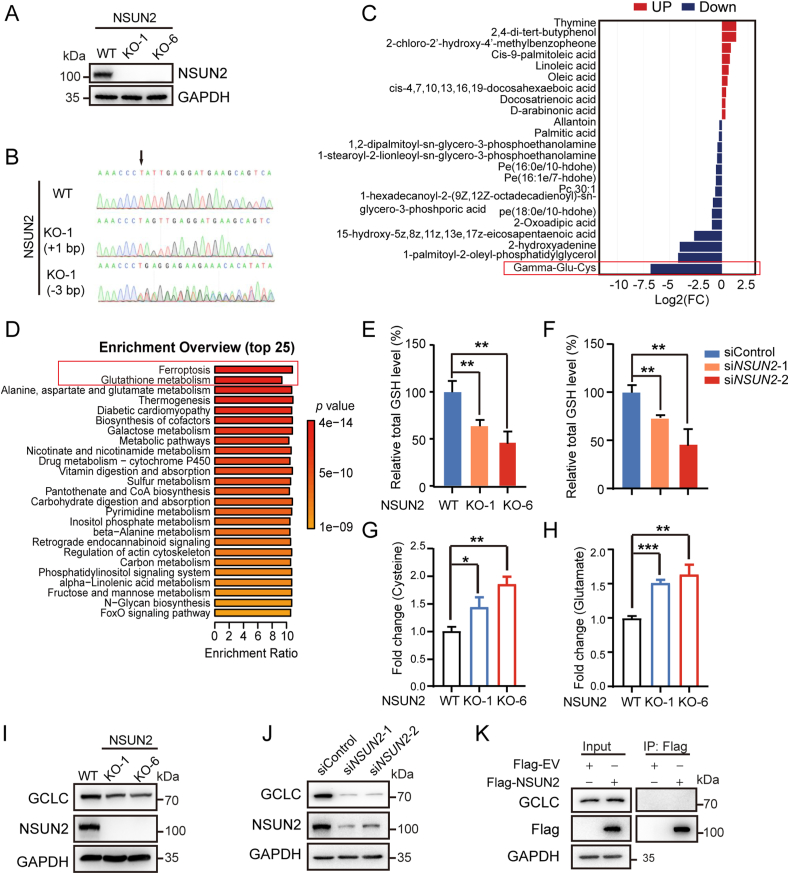
Intracellular total GSH level was significantly reduced after NSUN2 knockout in MKN45 cells. (A) The CRISPR/Cas9 system was used to create MKN45 NSUN2 knockout (KO) cells. The Western blot identified two monoclonals (KO clones #1 and #6). (B) Sanger sequencing of NSUN2 in normal MKN45 (WT) and mutant MKN45 cells (NSUN2 KO). Arrowhead indicated mutation consisting of a 1 bp insertion and a 3 bp deletion. (C) The differential metabolites between WT and NSUN2 KO MKN45 cells by untargeted metabolomics analysis (APTBIO, Shanghai, China). (D) Metabolite Set Enrichment Analysis (MSEA) between WT and NSUN2 KO cells, and summary plot for MSEA is ranked according to Holm *p*-value. (E and F) NSUN2 knockout or knockdown resulted in reduced intracellular total GSH level in MKN45 cells. (G and H) Glu and Cys concentrations were significantly increased in NSUN2 knockout cells compared to NSUN2 WT MKN45 cells. (I)Western blotting analysis of the protein expression level of GCLC after NSUN2 knockout in MKN45 cells. (J) Western blotting analysis of the protein expression level of GCLC after NSUN2 knockdown in MKN45 cells. (K) Co-immunoprecipitation (Co-IP) and Western blot analysis of HEK293 cells were transfected with Flag-NSUN2 for 48 h, and then endogenous GCLC protein was determined in Flag immunoprecipitates with anti-GCLC antibody by Western blot. The data represent the mean ± SD from three independent experiments. Statistical analysis by paired Student's *t*-test or two-way ANOVA. ∗P < 0.05, ∗∗P < 0.01, ∗∗∗P < 0.001.

To dissect the biological function of NSUN2 lactylation in tumor cells, NSUN2 was knocked out in MKN45 gastric cancer cells using CRISPR/Cas9 system (Fig. 2A and B), and NSUN2-null cells were subjected to untargeted metabolomics analysis (APTBIO, Shanghai, China) using vector-alone cells as control. The results revealed that gamma-glutamyl cysteine (γ-glu-cys), a substrate for the synthesis of glutathione, was lower in NSUN2 KO cells than that in WT cells (Fig. 2C). Metabolite Set Enrichment Analysis (MSEA) showed that the top two biological activities affected by NSUN2 deficiency are glutathione (GSH) synthesis and ferroptosis sensitivity (Fig. 2D). In support, intracellular total GSH level was significantly reduced after NSUN2 knockout or knockdown (Fig. 2E and F). Meanwhile, the levels of Glu and Cys were significantly increased in NSUN2 knockout MKN45 cells compared to control (Fig. 2G and H).

It is well-established that synthesis of γ-glu-cys (γ-GC) is catalyzed by a rate-limiting enzyme, glutamate cysteine ligase (GCL), composed of the main catalyzing subunit GCLC and the modifier subunit GCLM [41]. We then determined whether the major subunit of GCLC was the potential target of NSUN2. The results showed that NSUN2 KO or siRNA-mediated knock-down in MKN45 cells decreases GCLC protein expression levels (Fig. 2I and J), while co-IP assay for interaction of NSUN2 with GCLC and cycloheximide (CHX) treatment assay for GCLC protein stability yielded negative results (Fig. 2K and S2). In addition, we found that NSUN2 knockout has no effect on protein and mRNA levels of GCLM (Fig. S3). These findings suggest that lower GCLC protein level in NSUN2 KO cells might be attributed to the decreased GCLC mRNA, instead of proteasome pathway-mediated protein degradation.

**Fig. 3:**
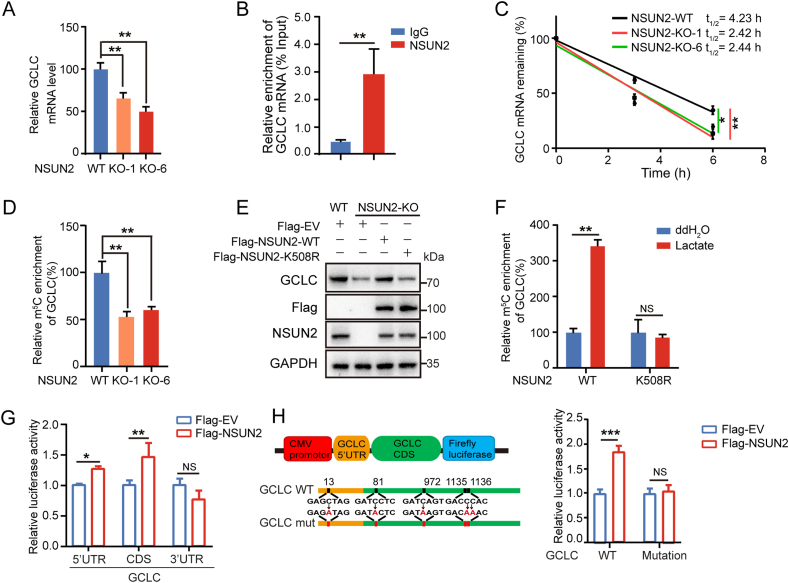
Lactylation of NSUN2 promotes GCLC accumulation via m5C modification on GCLC mRNA. (A) qRT-PCR analysis of GCLC mRNA expression level after NSUN2 knockout in MKN45 cells. (B) RNA immunoprecipitation of GCLC mRNA was carried out in MKN45 cells using anti-NSUN2 antibody, with IgG as the control. (C) Stability analysis of GCLC mRNA in WT or NSUN2 KO MKN45 cells (clone #1 and #6) with treatment of actinomycin D (ActD) for 0, 3 and 6 h. (D) The purified RNA was immunoprecipitated by anti-m^5^C antibody and the m^5^C level of GCLC in WT or NSUN2 KO MKN45 cells (clone #1 and #6) was analyzed by qRT-PCR. (E) The NSUN2 KO cells were transfected with Flag-NSUN2-WT or Flag-NSUN2-K508R plasmids for 48 h and harvested. Western blot analysis of the protein expression level of GCLC in indicated cells. (F) The NSUN2 KO cells were transfected with Flag-NSUN2-WT or Flag-NSUN2-K508R plasmids for 36 h and then treated with 20 mM lactate for 24 h. Methylated RNA immunopreciptitation (MeRIP) analysis of GCLC m^5^C level under lactate treatment. (G) Relative luciferase activity of 5′-UTR, CDS and 3′-UTR of GCLC reporter vectors in NSUN2 KO cells transfected with Flag-EV or Flag-NSUN2-WT. (H) Relative luciferase activity of GCLC-WT or GCLC-mutation (5-UTR mutation: C13A; CDS mutations: C81A, C972A, C1135A, C1136A) reporter vectors in NSUN2 KO cells transfected with Flag-EV or Flag-NSUN2-WT. The data represent the mean ± SD from three independent experiments. Statistical analysis by paired Student's *t*-test or two-way ANOVA. ∗*P* < 0.05. ∗∗*P* < 0.01. NS: not significant.

NSUN2 is a nuclear methyltransferase and responsible for the m5C modification in RNA [16]. We then tried to determine whether NSUN2 lactylation could enhance GCLC m^5^C-modification and mRNA stability. The results showed that NSUN2 KO in MKN45 cells markedly decreases GCLC mRNA expression levels (Fig. 3A). We then employed RNA immunoprecipitation (RIP) assay to test the physical interaction between NSUN2 protein and GCLC mRNA using NSUN2 antibody. The endogenous NSUN2 protein was shown to bind to native GCLC mRNA (Fig. 3B). To further investigate whether NSUN2 regulates the stabilization of GCLC, the half-life of GCLC mRNA was examined in NSUN2 WT and KO MKN45 cells by treatment with RNA synthesis inhibitor actinomycin D (ActD). The result demonstrated that NSUN2 KO significantly decreases the half-life of GCLC mRNA (Fig. 3C).

To determine whether NSUN2 regulates GCLC mRNA level through affecting its m^5^C modification, m^5^C-RIP-qPCR was performed to analyze the m^5^C-methylated GCLC mRNA levels in NSUN2 WT and KO MKN45 cells. The result showed that NSUN2 KO significantly decreases the endogenous level of m^5^C methylated GCLC mRNA in relative to WT cells (Fig. 3D). Meanwhile, the reduced GCLC protein level in NSUN2 KO cells could be recovered by WT NSUN2, but not its K508R lactylation mutant (Fig. 3E), suggesting that NSUN2 lactylation promotes GCLC expression through catalyzing its m^5^C modification. To validate this speculation, WT or K508R NSUN2 was transiently expressed in NSUN2 KO MKN45 cells, followed by the treatment with 20 mM lactate for 24 h and m^5^C-RIP-qPCR assay. The result revealed that lactate induces a significantly higher level of m^5^C methylation on endogenous GCLC mRNA in WT NSUN2 reconstituted cells, while this effect was not observed in K508R mutant cells (Fig. 3F). Collectively, these data support that lactate-derived lactylation of NSUN2 facilitates GCLC mRNA expression in an m^5^C-dependent manner.

To identify the m^5^C modification sites on GCLC mRNA, we first constructed pGL6-derived reporters bearing different GCLC domains of CDS (coding sequence), 5′UTR and 3′UTR. In consistent with the previous findings that NSUN2 stabilizes the mRNA targets through enhancing m5C modification [17], NSUN2 overexpression in NSUN2 KO cells significantly increased the luciferase activity of GCLC CDS and 5′ UTR reporters, but not 3′UTR (Fig. 3G), indicating that the methylation sites were most likely located at the CDS and 5′UTR of GCLC. Previous study using bisulfite sequencing assays has revealed the m^5^C methylation pattern with primary distribution in the CDS of the mRNA transcript and the existence of one methylated cytosine C13A in the 5′ UTR [16]. We also predicted the possible m^5^C modification sites on GCLC CDS region using m^5^C Finder (http://www.rnanut.net/rnam5cfinder/) and iRNA-m^5^C (http://lin-group.cn/server/iRNA-m5C/service.html). By integrating the above results, luciferase reporter plasmids, including wild-type (GCLC-WT) and m^5^C mutants (GCLC-mut), were constructed and co-transfected into NSUN2 KO cells with pRL-TK and Flag-NSUN2/Flag-CMV. The dual-luciferase assay results revealed that NSUN2 markedly increases the luciferase activity of GCLC-WT, but the effect is substantially decreased in the mutants (5′UTR: C13A and CDS: C81A, C972A, C1135A, and C1136A) (Fig. S4). In support, the induction of luciferase activity by WT NSUN2 was totally lost in the GCLC mutated with the above five sites (Fig. 3H), suggesting that these five cytosines are the main methylation sites by NSUN2. Moreover, these data strongly support that NSUN2-mediated m5C modification on GCLC mRNA is critical for GCLC mRNA stability.

### 3.3 Lactate drived lactylation of NSUN2 enhances ferroptosis resistance dependent of GCLC

**Fig. 4:**
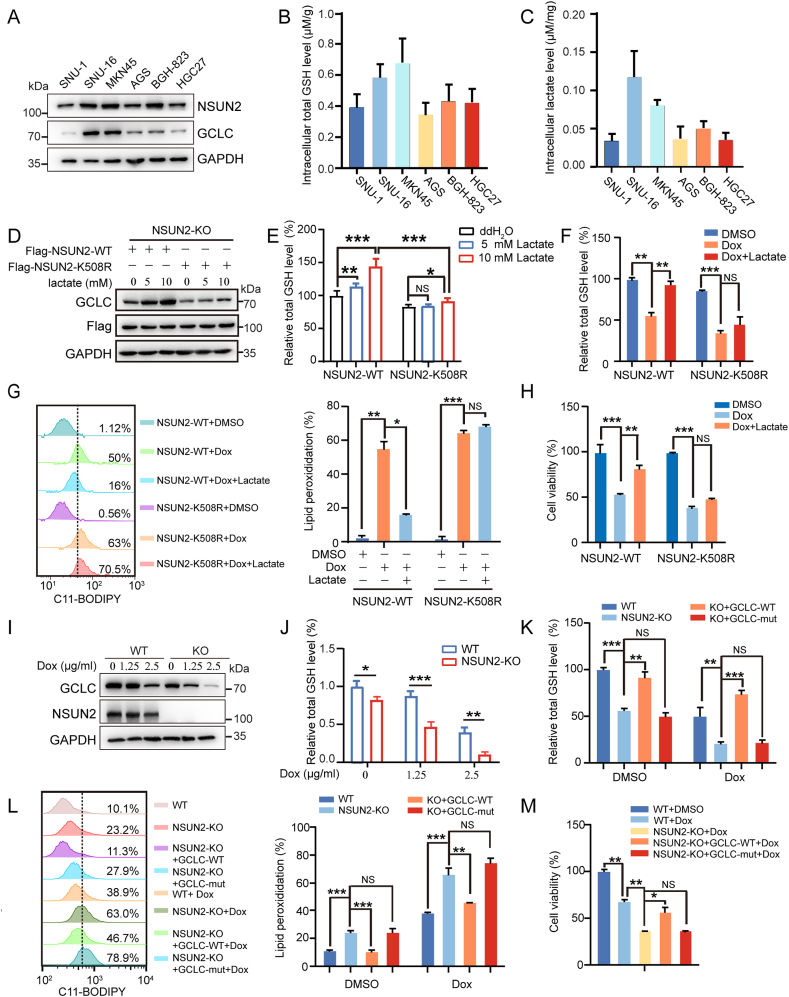
Lactate-mediated lactylation of NSUN2 induces ferroptosis resistance dependent of GCLC. (A) Expression levels of NSUN2 and GCLC were detected by Western blot in six gastric cancer cell lines (SNU-1, SNU-16, MKN45, AGS, BGH-823 and HGC27). (B) The intracellular total GSH levels were measured by Total Glutathione Assay Kit in six gastric cancer cell lines. (C) The intracellular lactate levels were measured by l-lactate assay kit in six gastric cancer cell lines. (D and E) NSUN2 KO MKN45 cells transfected with the NSUN2 WT or K508R were treated with 5 or 10 mM lactate. Indicated proteins and total GSH level were measured. (F–H) NSUN2 KO MKN45 cells were transfected with NSUN2 WT or K508R plasmids. (F) The indicated cells were pretreated with 10 mM lactate for 12 h, and then treated with 2.5 μg/ml Dox for 12 h. The total GSH level were measured. (G) Indicated cells were pretreated with 10 mM lactate for 12 h and then treated with 5 μg/ml Dox for 5 h. The lipid peroxidation level was assessed. (H) Indicated cells were pretreated with 10 mM lactate for 12 h, and then treated with 1.25 μg/ml Dox for 24 h. The viability of indicated cells were measured. (I and J) The WT and NSUN2 KO cells were treated with 1.25 or 2.5 μg/ml Dox for 12 h. GCLC protein expression and total GSH levels were measured. (K–M) The WT or NSUN2 KO cells were transfected with Flag-GCLC-WT or Flag-GCLC-mutation for 36 h. (K and L) Indicated cells were treated with 2.5 μg/ml Dox for 12 h. The total GSH and lipid peroxidation levels were assessed. (M) Indicated cells were treated with 1.25 μg/ml Dox for 24 h. The viability of indicated cells were measured. The data represent the mean ± SD from three independent experiments. Statistical analysis by paired Student's *t*-test or two-way ANOVA. ∗*P* < 0.05, ∗∗*P* < 0.01, ∗∗∗*P* < 0.001, NS: not significant.

As GCLC-catalyzed GSH synthesis plays a key role in redox homeostasis and ferroptosis sensitivity [42,43], we further evaluated whether NSUN2 lactylation regulates GCLC-dependent sensitivity to ferroptosis in cancer cells. We first analyzed the protein levels of NSUN2 and GCLC in six gastric cancer cell lines by western blotting analysis, and found a relatively higher level of NSUN2 in all cell lines, except SNU-1. Intriguingly, two cell lines including SNU-16 and MKN45, present much higher GCLC protein levels in relative to other four tumor cell lines (Fig. 4A), and both the intracellular lactate and total GSH levels were also higher (Fig. 4B and C). This data illustrated a well-correlation between lactate level and GCLC expression/GSH synthesis, pointing to a potential significance of lactylation-modulated enzymatic activity for NSUN2. To prove it, NSUN2 KO MKN45 cells were transfected with NSUN2 WT or K508R mutant followed by the treatment with 5 mM or 10 mM lactate for 24 h. The results showed that upon lactate treatment, NSUN2 WT, but not K508R mutant, reconstituted cells display a substantially increased GCLC protein expression and meanwhile, a significantly enhanced intracellular total GSH level (Fig. 4D and E). Thus, higher lactate mediates the lactylation of NSUN2 which then promotes GCLC-dependent GSH synthesis.

To determine whether lactate-mediated NSUN2 lactylation contributes to the resistant phenotype to ferroptosis in cancer cells, WT NSUN2 or its K508R mutant was transfected into NSUN2 KO MKN45 cells, followed by sequential treatments with lactate and then Dox. The results showed that only Dox treatment significantly decreases the total GSH level (Fig. 4F) vs. increases lipid peroxidation level and ferroptotic cell death (Fig. 4G and H). Nevertheless, these effects were reversed by pretreatment with lactate in cell overexpressed with WT NSUN2, but not K508R mutant (Fig. 4F–G). It should be pointed out that our study primarily employed Dox treatment for ferroptosis induction mainly based on our findings that ferroptosis inhibitor Fer-1, instead of apoptosis inhibitor (Z-VAD-FMK), significantly reduced the Dox-induced cell death in NSUN2 WT or KO MKN45 cells (Fig. S5). In support, the result from Wang's group demonstrated that in a DOX-induced cardiotoxic mouse model, only ferroptosis inhibitor Fer-1, instead of other forms of cell death inhibitors, could significantly reduce the Dox-induced mortality [44]. Nevertheless, we also treated the cells with RSL3, a classical ferroptosis inducer, and consistent results as Dox treatment were also acquired (Figs. S5 and 6), suggesting that lactate-mediated NSUN2 lactylation could protect gastric cancer cells from Dox or RSL3-induced ferroptosis.

To determine whether NSUN2 lactylation-regulated ferroptosis depends on GCLC, we compared the GCLC protein expression and total GSH levels between NSUN2 WT and KO MKN45 cells with or without Dox treatment for 12 h. The results showed that even though both GCLC and total GSH were decreased in both cell types upon Dox treatment, the changes were more pronounced in NSUN2 KO cells, suggesting that NSUN2 null results in defective GCLC expression and GSH synthesis (Fig. 4I and J). To address how Dox decreases GCLC expression, we the examined the lactate level change under Dox treatment. The result showed that Dox (0.625, 1.25, 2.5 μg/ml) treatment in MKN45 cells significantly reduced the intracellular lactate level even at low concentration of Dox treatment (Fig. S7A). The global Kla abundance was also substantially decreased (Fig. S7B). In addition, lactylation level of NSUN2 under Dox treatment was substantially decreased (Fig. S7C). These results support that Dox treatment can decrease GCLC expression through inducing a reduced lactate level which consequently lessens the NSUN2 lactylation and further its catalyzing activity.

We next tested the significance of NSUN2-mediated m^5^C modification on GCLC in regulating the sensitivity to ferroptosis induction. The results showed that in relative to NSUN2 WT MKN45 cells, NSUN2 KO results in a significantly decreased total GSH level, with a more distinct change tendency under Dox treatment in relative to DMSO. Moreover, this change could be rescued by reconstitution in NSUN2 KO cells with WT GCLC, but not its five-cytosine C-A mutant (Fig. 4K). The protein levels were examined by western blotting (Fig. S8). Consistently, the increased lipid peroxidation level together with decreased cell viability in NSUN2 KO cells upon Dox treatment could be reversed by WT GCLC, instead of its five C-A mutant (Fig. 4L and M). Overall, these findings imply that GCLC is an indispensable downstream effector of lactylated NSUN2 in modulating ferroptosis resistance to promote cancer cell survival.

### 3.4 Identification of NAA10 as the lactyltransferase of NSUN2

**Fig. 5:**
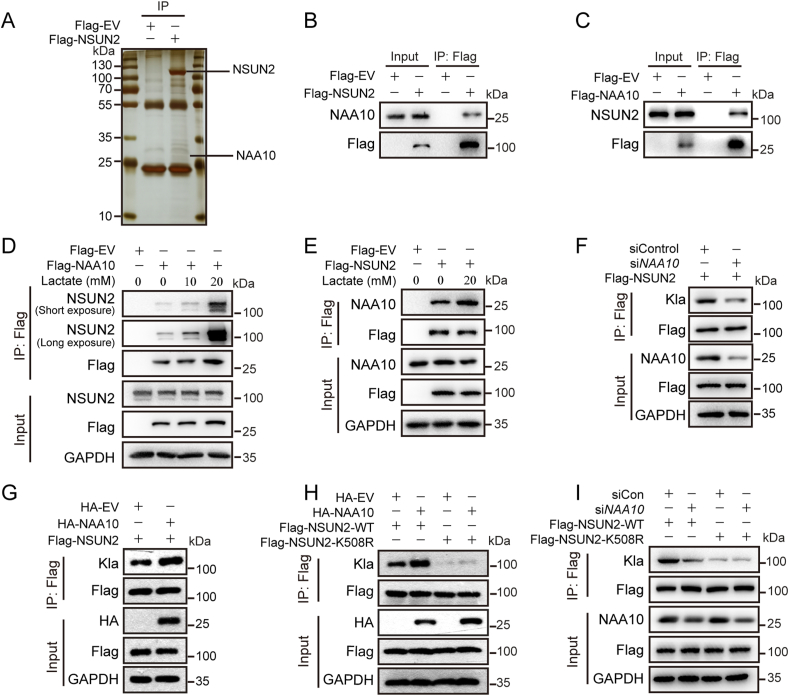
Identification of NAA10 as the NSUN2 lactyltransferase at site of K508. (A) Silver-staining of NSUN2 interacting proteins. Flag-NSUN2 were transfected into HEK293 cells for 48 h and cell lysate was immunoprecipitated with antibody against Flag M2 beads and analyzed by a Mass-Spectrometer. (B) Endogenous NAA10 protein was detected in anti-Flag immunoprecipitate from cell lysate of Flag-NSUN2 transfected HEK293 cells. Flag-EV was used for negative control. (C) Endogenous NSUN2 protein was verified in anti-Flag immunoprecipitate from cell lysate of HEK293 cells overexpressing Flag-NAA10 by western blotting. (D and E) The interaction between NSUN2 and NAA10 was enhanced under lactate treatment. (D) HEK293 cells were transfected with Flag-NAA10 for 48 h, and then treated with 10 mM or 20 mM lactate for 3 h. Endogenous NSUN2 protein was examined in Flag immunoprecipitates with anti-NSUN2 antibody by western blotting. (E) HEK293 cells were transfected with Flag-NSUN2 for 48 h, and then treated with 20 mM lactate for 3 h. Endogenous NAA10 protein was determined in Flag immunoprecipitates with anti-NAA10 antibody by western blotting. (F) MKN45 cells were transfected with Flag-NSUN2 followed by treatment with si*NAA10* or siControl. NSUN2 lactylation level was examined in Flag immunoprecipitates with anti-lactyllysine antibody by western blotting. (G) Increased lactylation level of NSUN2 in HEK293 cells overexpressing HA-NAA10. HEK293 cells were transfected with Flag-NSUN2 and HA-NAA10 or HA-EV for 48 h. NSUN2 lactylation level was examined in Flag immunoprecipitates with anti-lactyllysine antibody by western blotting. (H) NSUN2 was lactylated by NAA10 at K508. HEK293 cells were co-transfected with indicated expression plasmids and harvested after 48 h. The lactylation level of NSUN2-WT or K508R upon NAA10 overexpression were determined by western blotting with anti-lactyllysine antibody. (I) MKN45 cells were transfected with Flag-NSUN2-WT or Flag-NSUN2-K508R followed by treatment with si*NAA10* or siControl for 48 h. NSUN2 lactylation level was examined in Flag immunoprecipitates with anti-lactyllysine antibody by western blotting.

To search for the potential enzymes catalyzing the lactylation modification of NSUN2, tandem affinity purification coupled with mass spectrometry was employed on the immunoprecipitates from Flag-NSUN2 overexpressed HEK293 cells (Fig. 5A and Table S1). Several potential NSUN2-interacting proteins, including N-terminal acetyltransferase NAA10 [45], were identified. We next examined the physical interaction of NAA10 with NSUN2 by a coimmunoprecipitation (co-IP) assay, and observed the presence of endogenous NAA10 in Flag-NSUN2 pull-down complex using anti-Flag antibody (Fig. 5B). Likewise, endogenous NSUN2 was found to bind to Flag-NAA10, but not Flag tag (Fig. 5C). The association between NSUN2 and NAA10 under lactate treatment was further examined. Flag-NAA10 was expressed in MKN45 cells with or without lactate treatment for 24 h, and the result showed a much strengthened interaction between these two proteins upon lactate treatment (Fig. 5D and E).

To determine whether NAA10 acts as a writer of NSUN2 lactylation, NAA10 was knockdown in Flag-NSUN2 overexpressed MKN45 cells followed by co-IP using anti-Flag antibody. NSUN2 Kla detected with anti-lactyllysine antibody was observed to be substantially decreased (Fig. 5F). Likewise, NAA10 overexpression substantially increased NSUN2 Kla level in HEK293 cells (Fig. 5G). We further determined whether NSUN2 K508 is the major acting site of NAA10 by transfecting NSUN2-WT or K508R mutant plasmids in HEK293 cells, followed by HA-NAA10 overexpression or knockdown by NAA10 small interfering RNA (siNAA10). The results demonstrated that K508R mutation leads to a nearly lost effect of NAA10 on NSUN2 Kla level (Fig. 5H and I). These findings provide strong evidence that NAA10 possesses lactyltransferase activity catalyzing NSUN2 lactylation at K508.

### 3.5 NAA10 protects gastric cancer cells from ferroptosis through NSUN2 lactylation-dependent GCLC expression

**Fig. 6:**
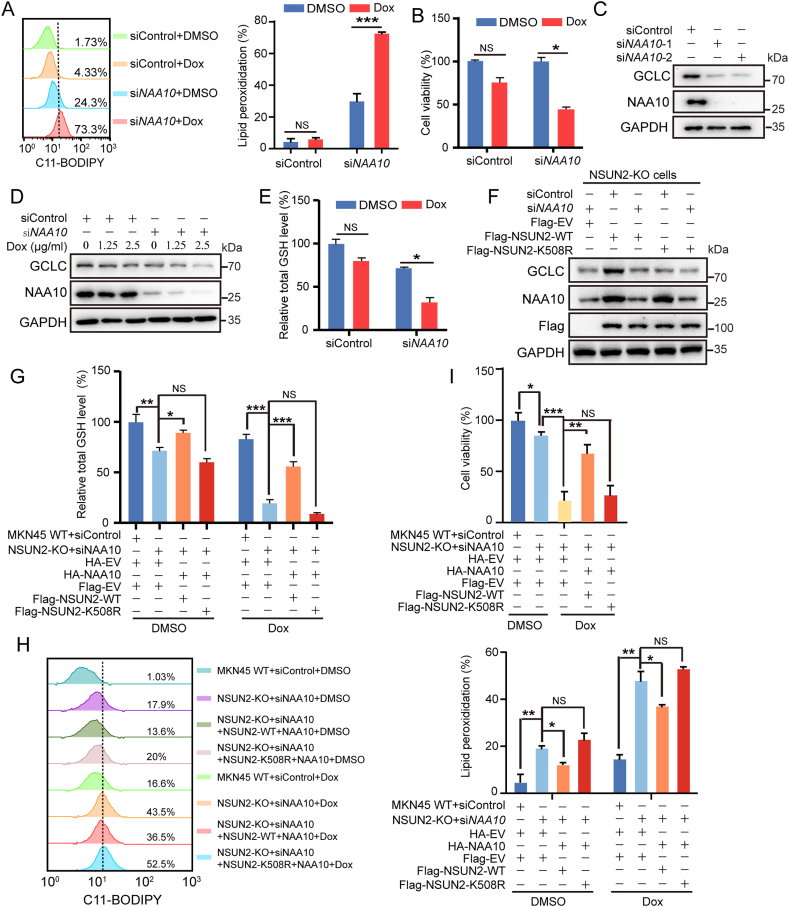
NAA10 protects gastric cancer cells from ferroptosis through activation of NSUN2-GCLC pathway. (A) The MKN45 cells were transfected with siControl or *NAA10* siRNA. Indicated cells were treated with 2.5 μg/ml Dox for 12 h. The lipid peroxidation level was assessed. (B) The viability of indicated cells with treatment with 1.25 μg/ml Dox for 24 h were measured. (C) Western blotting analysis of GCLC protein expression in indicated cells. (D) The MKN45 cells were transfected with siControl or *NAA10* siRNA followed by the treatment of 1.25 or 2.5 μg/ml Dox for 12 h. The GCLC protein expression was determined by western blotting. (E) The MKN45 cells were transfected with siControl or *NAA10* siRNA. Indicated cells were treated with 2.5 μg/ml Dox for 12 h. Changes in the intercellular total GSH level were measured. (F) The NSUN2 KO MKN45 cells were transfected with Flag-NSUN2-WT or Flag-NSUN2-K508R for 24 h, and transfected with siControl or *NAA10* siRNA. The GCLC protein expression were determined by western blotting. (G–I) The NSUN2 KO MKN45 cells were transfected with siControl or *NAA10* siRNA for 24 h and then transfected with Flag-NSUN2-WT + HA-NAA10 or Flag-NSUN2-K508R + HA-NAA10 for 24 h. (G and H) Indicated cells were treated with 2.5 μg/ml Dox for 12 h. The intercellular total GSH and lipid peroxidation level was assessed. (I) Indicated cells were treated with 1.25 μg/ml Dox for 24 h. The viability of indicated cells were measured. The data represent the mean ± SD from three independent experiments. Statistical analysis by paired Student's *t*-test or two-way ANOVA. ∗*P* < 0.05, ∗∗*P* < 0.01, ∗∗∗*P* < 0.001, NS, not significant.

We next tried to determine whether NAA10 expression regulates ferroptosis sensitivity in cancer cells. NAA10 was knocked down in MKN45 cells using siNAA10, and results showed that compared to the siRNA control, Dox treatment in the knockdown cells significantly increased the lipid peroxidation level (Fig. 6A) and meanwhile decreased the cell viability (Fig. 6B). Although cell viability of siControl cells also decreased upon dox treatment, but was at a less extent and without statistical significance. This finding demonstrates that NAA10 regulates ferroptosis in cancer cells.

We next determined the critical role of NSUN2 lactylation-GCLC-GSH signaling axis in NAA10-regulated ferroptosis. We first examined the GCLC and total GSH levels under *NAA10* silencing. As shown in Fig. 6C, siRNA-mediated *NAA10* knockdown in MKN45 cells substantially decreased GCLC expression level. Expecially under dox treatment, GCLC protein level was more obviously decreased in relative to siControl (Fig. 6D). In support, GSH synthesis was significantly decreased in si*NAA10* cells under Dox (Fig. 6E), suggesting that NAA10 regulates intracellular GCLC and total GSH levels, which is most like implicated in NAA10-regulated ferroptosis sensitivity.

We next determined whether NAA10 regulated GCLC level depends on NSUN2 lactylation. WT NSUN2 or its lactylation mutant K508R plasmids were transfected into NSUN2 KO MKN45 cells followed by *NAA10* knockdown by si*NAA10* (Fig. 6F). The results showed that NAA10 depletion substantially decreases GCLC protein level in WT NSUN2 reconstituted cells, but not in K508R cells, suggesting that NAA10 regulates GCLC expression through NSUN2 lactylation.

**Fig. 7:**
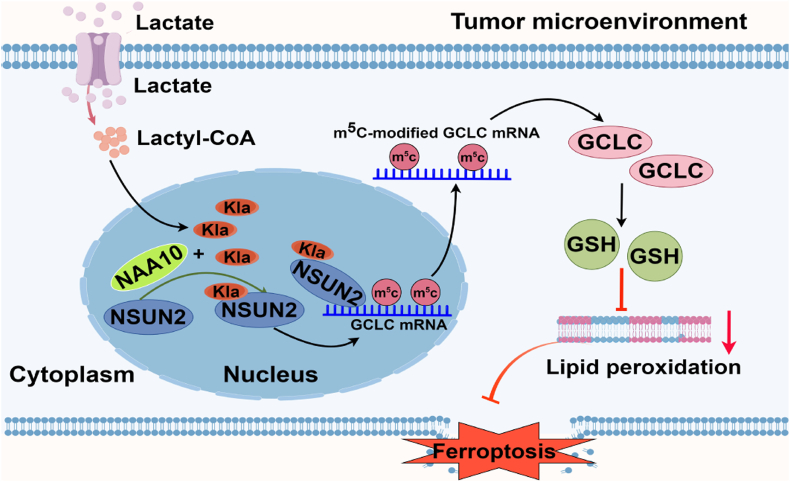
NSUN2 lactylation promotes cancer cell survival through enhancing GCLC-dependent glutathione synthesis. Lactate treatment substantially enhances NSUN2 interaction with lactyltransferase NAA10, leading to elevated NSUN2 lactylation and enzymatic activation, which then targets GCLC mRNA to promote m^5^C formation and mRNA stability that induces GSH synthesis.

To further explore the significance of NSUN2 lactylation in NAA10 regulated ferroptosis, NSUN2 KO MKN45 cells were depleted for NAA10 expression with si*NAA10* for 24 h and then reconstituted with NAA10 and WT NSUN2/K508R for 24 h, followed by Dox treatment. The protein expression levels were examined (Fig. S9). The results showed that under both DMSO and Dox treatments, cells with NSUN2 KO and NAA10 knockdown significantly decreased the intracellular total GSH level (Fig. 6G), and markedly increased the lipid peroxidation levels in relative to NSUN2 WT + siControl group (17.9 % vs. 1.03 % for DMSO and 43.5 % vs. 16.6 % for Dox) (Fig. 6H). Meanwhile, cell viability was significantly decreased in both treatment conditions in NSUN2 KO and NAA10 knockdown cells (Fig. 6I). However, in comparison to DMSO, the changes in total GSH, lipid peroxidation and cell viability were more pronounced under Dox treatment condition. More importantly, all these altered phenotypes can be rescued by re-expression of NAA10 with WT NSUN2, but not NAA10+K508R mutant (Fig. 6G–I). These results clearly indicate that NSUN2 lactylation catalyzed by NAA10 promotes NSUN2 catalyzing activity, which mediates the activation of NSUN2-GCLC-GSH signaling pathway to protect gastric cancer cells from ferroptosis (see Fig. 7).

## 4 Discussion

Abnormal high level of extracellular lactate is the characteristic feature of TME and has been associated with the increased cellular level of reactive oxygen species (ROS) [1,46,47]. Lactate, a common metabolite in the tumor microenvironment [48], is a pleiotropic signaling molecule and can mediate lactylation modification on histone or non-histone proteins, leading to the regulation of various cellular processes including DNA repair [6], autophagy [49], oxidative damage [50] and ferroptosis [3]. Along with cancer progression, key metabolites and enzymes are temporally and functionally regulated, with key feature of metabolic heterogeneity in the TME [1,45,51]. However, how cancer cells adapt to the metabolic plasticity, such as the continuously changed levels of lactate, to promote their survival and metastasis remain unclear. Recent reports have shown that ectopic expression of NSUN2 drives ferroptotic resistance in cancer cells through modulating a list of target genes, such as SLC7A11, NRF2 and GPX4 [22,52,53]. Moreover, lactylation modification has been discovered to regulate ferroptosis through targeting histone and non-histone proteins (METTL3, HIF1A, MDH2, tau) implicated in various pathological conditions, including sepsis-associated lung injury, prostate cancer, myocardial ischemia-reperfusion injury and Alzheimer's disease [[54], [55], [56], [57]]. However, whether NSUN2 function is regulated by lactylation which further affects ferroptotic resistance phenotype in cancer cells is still unclear. In this study, we provide strong evidence that lactate could induce lactylation of NSUN2 through enhancing its association with NAA10 who was demonstrated to possess lactyltransferase activity. The NSUN2 lactylation promotes its enzymatic activity to catalyze m^5^C modification on target GCLC mRNA, leading to upregulated GCLC expression and GSH synthesis rendering cancer cells resistant to ferroptosis. Overall, lactate-mediated lactylation of NSUN2 represents a critical PTM mechanism to regulate tumor cell survival under lactate stress.

N6-methyladenosine (m^6^A) and 5-methylcytosine (m^5^C) represent the two major types of RNA methylation, whose writers or readers have been shown to be functionally regulated by the lactylation-dependent mechanism. For instance, RNA m^6^A methyltransferase METTL3 was upregulated by histone lactylation (H3K18) in tumor microenvironment, and moreover, METTL3 lactylation at sites of K281 and K345 enhances its ability for target RNA capturing and m6A modification deposition on target mRNAs, such as Jak1, potently inducing the immunosuppressive functions of tumor-infiltrating myeloid cells (TIMs) [11]. In addition, expression of YTH N6-methyladenosine RNA Binding Protein F2 (YTHDF2) was facilitated by histone lactylation to drive oncogenesis in ocular melanomas [58]. Just like m^6^A, m^5^C is another important type of mRNA methylation modification catalyzed by NOP2/Sun domain family member 2 (NSUN2) and closely associated with various biological processes, including RNA metabolism, cell cycle control, cell proliferation and differentiation, and development through affecting target mRNA degradation or translation [16,24]. Our study demonstrates that GCLC mRNA, the catalyzing enzyme subunit of GSH synthesis, is the special target of lactylated NSUN2, through which cancer cells maintain a redox homeostasis under acidic TME.

Functional studies of Kla-modified proteins or their related pathways require the knowledge of their catalytic enzymes (writers and erasers) and substrates. The acyltransferase of p300, KAT8 and AARS1 have been suggested to be potential Writers for lactylation, as evidenced by their capability to increase histone Kla levels in cells [4,[59], [60], [61]]. In parallel, *in vitro* screening has revealed that the de-acetylases of HDAC1-3 and SIRT1-3 have delactylase activity to remove Kla from histones [62]. However, which delactylase and lactytransferase that process the lactylation or de-lactylation of NSUN2 remains unclear. Our study identified NAA10 with lactytransferase activity specifically lactylating NSUN2. NAA10 as the catalytic subunit of N-acetyltransferase A (NatA) has been reported to acetylate lysine residues of several substrates, including HIF-1α, Runx2, β-catenin, myosin light-chain kinase and androgen receptor [63,64]. However, there is no report regarding its lactytransferase activity. We firstly found that NAA10 knockdown reduces the overall lactylation level, indicating that NAA10 is a writer of global protein lactylation (Fig. S10). Specifically, lactate treatment promotes the direct association between NAA10 and NSUN2, leading to NSUN2 lactylation and consequently enhanced catalyzing activity. Even though acetylation modification also exists on NSUN2, lactate treatment only remarkably increases the lactylation modification, instead of acetylation level of NSUN2 (Fig. S11A). Moreover, mutation of NAA10-targeted lysine site (K508R) on NSUN2 failed to show any effect on NSUN2 acetylation (Fig. S11B), suggesting that NAA10 regulates NSUN2 activity mainly through mediating its lactylation modification.

Ferroptosis is a new type of programmed cell death driven by iron-dependent phospholipid peroxidation [31]. During ferroptosis, two fundamental processes trigger oxidative membrane damage: iron accumulation and lipid peroxidation [65], and the *xc*^*-*^-GSH-GPX4 antioxidant system plays a central role in protecting cells from ferroptosis. GSH is the primary enzymatic antioxidant fighting against lipid peroxidation in organisms [66], and *de novo* synthesized by GCL (glutamate-cysteine ligase) containing the catalytic subunit GCLC and modifier subunit GCLM. Our metabolite analysis and GSH measurement assay identified GCLC-GSH pathway as the direct target of NSUN2. Our results provide further mechanistic role of NSUN2 in ferroptosis through inducing GCLC m^5^C modification and further mRNA stability.

SLC7A11 (xCT) is a multi-pass transmembrane protein mediating the cystine/glutamate antiporter activity, and its protective role in counteracting oxidative stress-induced cell death is well-established [67,68]. The ferroptosis inducer RSL3 has been shown to either promotes SLC7A11 expression through activating transcription factor ATF4, or inhibits its expression through the nuclear factor kappa-B (NF-κB) activation [69,70]. However, one more recent report demonstrated that cancer cells with high overexpression of SLC7A11 are particularly vulnerable to oxidative stress due to the excessive cystine accumulation, suggesting that proper high level of SLC7A11 expression is crucial for cancer cell survival under oxidative stress [71]. In our study, we found that SLC7A11 was one of the targets of NSUN2 lactylation, and RSL3 treatment decreases SLC7A11 expression in MKN45 cells (Fig. S12). Moreover, both intracellular lactate and NSUN2 lactylation levels were substantially reduced upon RSL3 treatment (Figs. S13A and B). Since SLC7A11 is the target gene of NSUN2 through m^5^C modification [22], our findings support that RSL3 treatment inhibits SLC7A11 expression most likely through decreasing lactate production and NSUN2 lactylation.

In summary, lysine lactylation (Kla), as a novel PTM, provides a new perspective on the mechanism by which lactate functions [4]. Our study provides the compelling evidence that lactate can serve as a signaling molecule to lactylate and activate NSUN2 catalyzing m^5^C formation and maintaining mRNA stability of GCLC, by which cancer cells can avoid ferroptotic cell death under acidic TME. As such, specifically targeting lactylated NSUN2 by interfering its association with NAA10 may constitute a promising therapeutic approach in cancer treatment.

## CRediT authorship contribution statement

**Kaifeng Niu:** Writing – review & editing, Writing – original draft, Visualization, Validation, Supervision, Software, Resources, Project administration, Methodology, Investigation, Funding acquisition, Formal analysis, Data curation, Conceptualization. **Zixiang Chen:** Writing – review & editing, Writing – original draft, Methodology, Investigation, Formal analysis, Data curation, Conceptualization. **Mengge Li:** Writing – review & editing, Writing – original draft, Software, Project administration, Methodology, Formal analysis, Data curation, Conceptualization. **Guannan Ma:** Software, Methodology, Formal analysis, Data curation. **Yuchun Deng:** Visualization, Validation, Software, Resources, Methodology, Conceptualization. **Ji Zhang:** Software, Resources, Methodology, Formal analysis, Conceptualization. **Di Wei:** Software, Resources, Methodology, Formal analysis. **Jiaqi Wang:** Formal analysis, Conceptualization. **Yongliang Zhao:** Writing – review & editing, Writing – original draft, Supervision, Resources, Project administration, Investigation, Funding acquisition, Conceptualization.

## Funding

This work was supported by 10.13039/501100012166National Key R&D Program of China (grant no. 2019YFA0801702), the Innovative Research Group Program of 10.13039/501100001809National Science Foundation of China (grant no. 32121001), Assignment Form of Beijing Natural Science Foundation (International Scientists Project) (grant no. IS23079), the Strategic Priority Research Program of the 10.13039/501100002367Chinese Academy of Sciences (XDA0460403), 10.13039/501100012492Youth Innovation Promotion Association Foundation of 10.13039/501100002367Chinese Academy of Sciences (grant no. 2021096), China, and 10.13039/501100001809National Natural Science Foundation of China (grant no. 32000428).

## Declaration of competing interest

The authors declare that they have no known competing financial interests or personal relationships that could have appeared to influence the work reported in this paper.

## Figures and Tables

**figs1:**
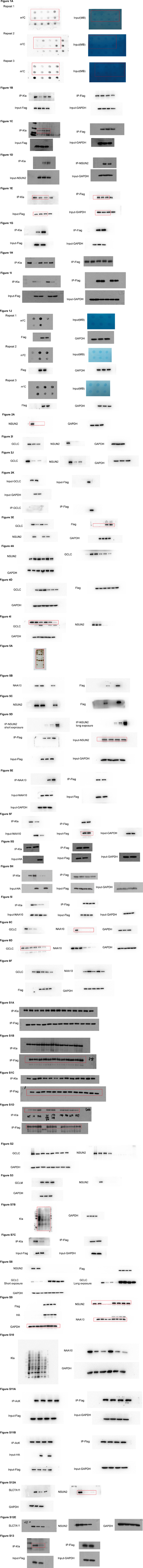


## Data Availability

Data will be made available on request.

## List of abbreviations

TME: Tumor Microenvironment
Kla: Lysine Lactylation
m^5^C: 5-methylcytosine
NSUN2: NOP2/Sun domain family member 2
HR: Homologous Recombination
iCCA: Clinical Intrahepatic Cholangiocarcinoma
PDAC: Pancreatic Ductal Adenocarcinoma
TTK: TTK protein kinase
LDH: Lactate Dehydrogenase
CBP: CREB Binding Protein
PTMs: Posttranslational Modifications
RCD: Regulated Cell Death
co-IP: co-immunoprecipitation
γ-glu-cys: gamma-glutamyl cysteine
GSH: Glutathione
GCL: Glutamate Cysteine Ligase
CHX: Cycloheximide
RIP: RNA Immunoprecipitation
ActD: Actinomycin D
