# Supplementary material for: NSUN2 lactylation drives cancer cell resistance to ferroptosis through enhancing GCLC-dependent glutathione synthesis
Source: Redox Biol. 2024 Dec 19;79:103479. doi: 10.1016/j.redox.2024.103479 (PMC11750563; doi:10.1016/j.redox.2024.103479)
Supplement: Multimedia component 1 [file mmc1.docx]

Supporting Information

**Title**

NSUN2 lactylation drives cancer cell resistance to ferroptosis through enhancing GCLC-dependent glutathione synthesis

Kaifeng Niu^1,2^*, Zixiang Chen^1,2^, Mengge Li^1,2,3^, Guannan Ma^4^, Yuchun Deng^1,2,3^, Ji Zhang^1,2,3^, Di Wei^1,2^, Jiaqi Wang^1,2,3,^ Yongliang Zhao^1,2,3^*

**Table S1.** Identification of NSUN2-interacting proteins by mass spectrometry.

| **Gene names** | **Proteins** | **Mass** | **Score** | **Intensity** |
| --- | --- | --- | --- | --- |
| NSUN2 | Q08J23 | 2587.1998 | 469.15 | 2.22E+09 |
| NAA10 | P41227 | 1078.6135 | 104.44 | 12097000 |


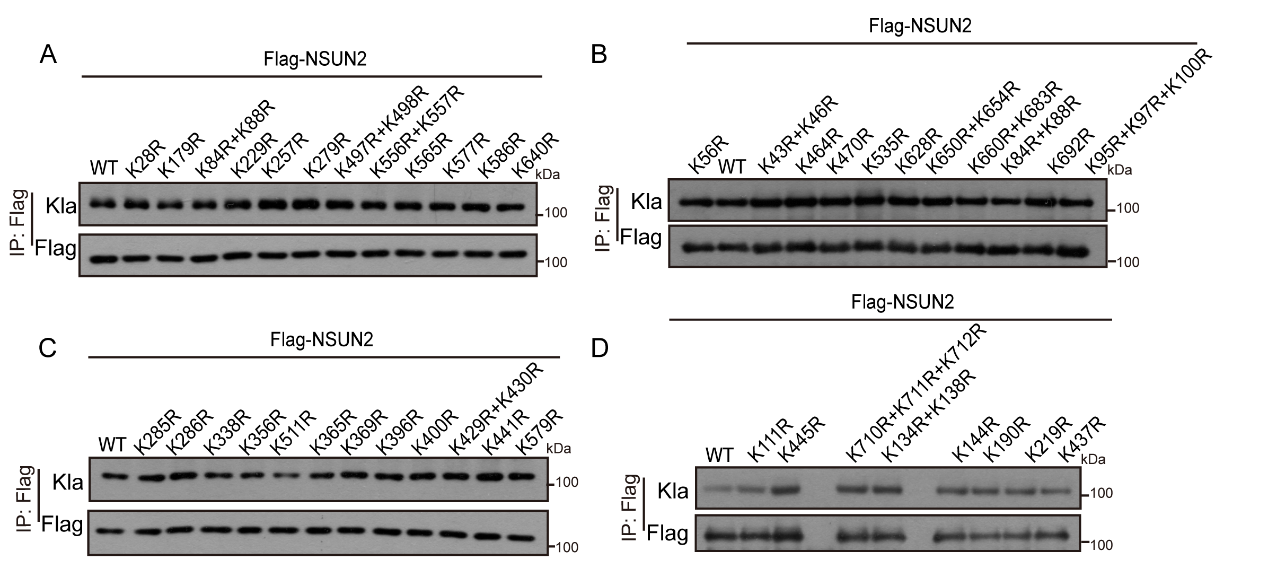


**Figure S1. Lysine site screening potentially lactylated on NSUN2.** HEK293 cells were co-transfected with individual lysine site-mutated Flag-NSUN2 for 36 h. Lactylation level of NSUN2 mutant was examined in Flag immnoprecipitates by western blotting with anti-lactyllysine antibody.


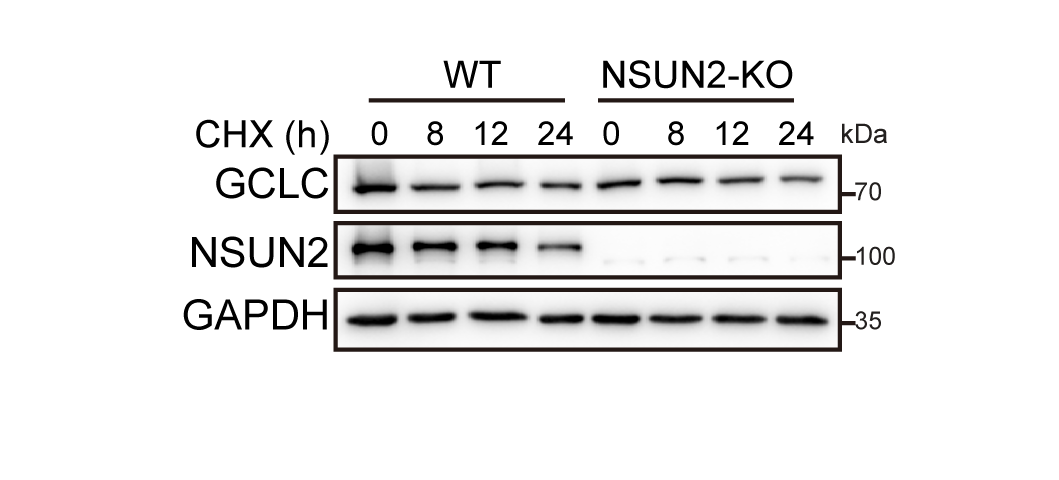


**Figure S2. The cycloheximide (CHX) treatment failed to alter the GCLC protein levels in both NSUN2 WT and KO MKN45 cells**. (A) WT and NSUN2 KO cells were treated with cycloheximide (CHX) for 8, 12 or 24 h and harvested. Western blot analysis of the protein expression level of GCLC in indicated cells.


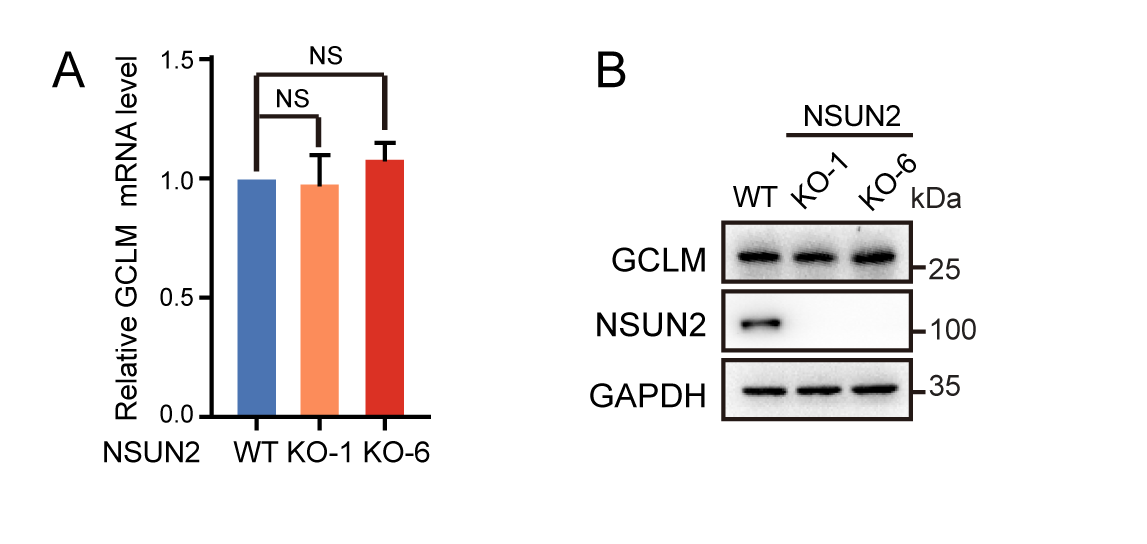


**Figure S3. The mRNA and protein levels of GCLM in NSUN2 wild type and knockout MKN45 cells.** (A) qRT-PCR quantification of GCLM mRNA level. (B) Western blotting analysis of the protein expression of GCLM. The data represent the mean±SD from three independent experiments. Statistical analysis by paired Student’s t test. NS: not significant.


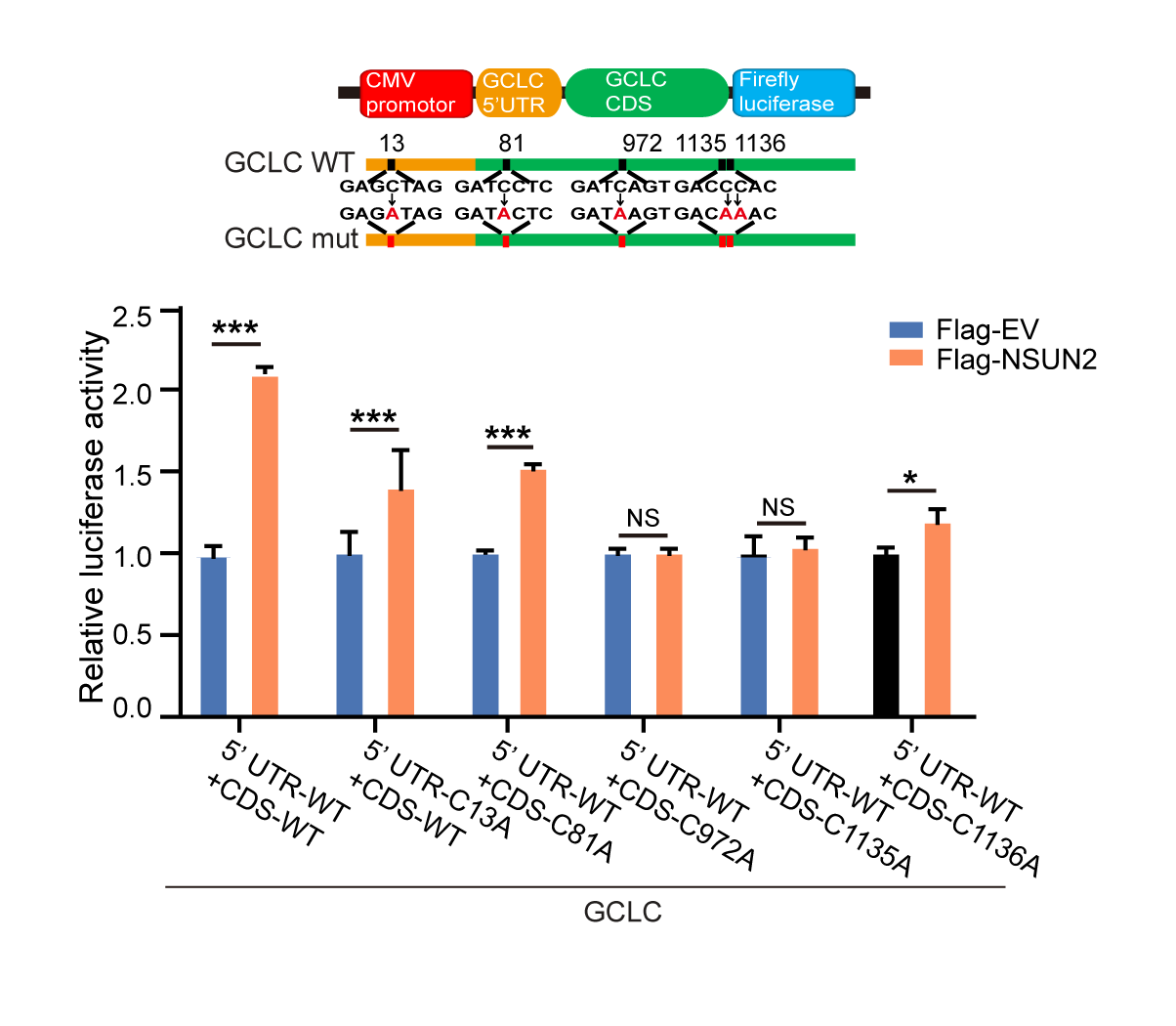


**Figure S4.** **The m^5^C sites screening on GCLC.** Relative luciferase activity of 5’UTR-WT + CDS-WT, 5’UTR-mutation (C13A) + CDSWT, 5’UTR-WT + CDS-mutations (C81A, C972A, C1135A, C1136A) of GCLC reporter vectors in NSUN2 KO cells transfected with Flag-EV or Flag-NSUN2-WT. n = 3 biological replicates. The data represent the mean ± SD from three independent experiments. Statistical analysis by two-way ANOVA.**P* < 0.05, ****P* < 0.001, NS not significant.

**
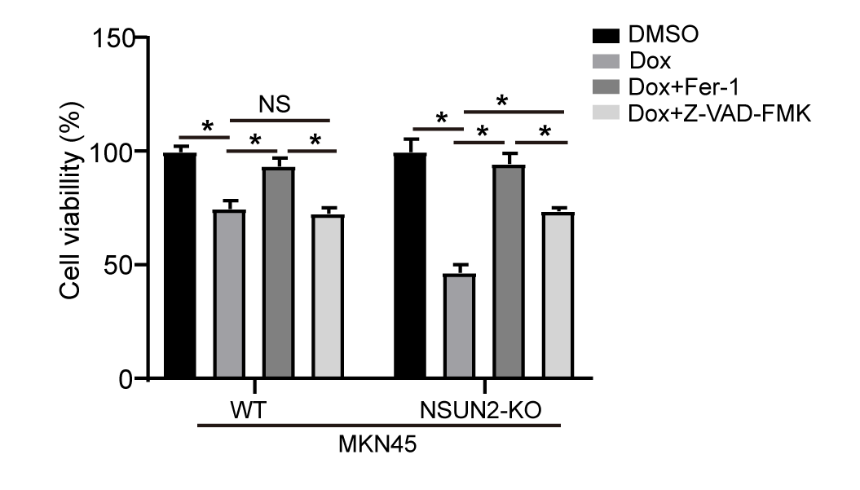
**

**Figure S5. Ferroptosis inhibitor fer-1 inhibits Dox induced cell death.** MKN45 WT and NSUN2-KO cells were pretreated with Fer-1 or Z-VAD-FMK for 12 h, then treated with 1.25 μg/ml Dox for 24 h. And cell death was determined by CCK-8 assay. The data represent the mean±SD from three independent experiments. Statistical analysis by two-way ANOVA. *P < 0.05, NS, not significant.


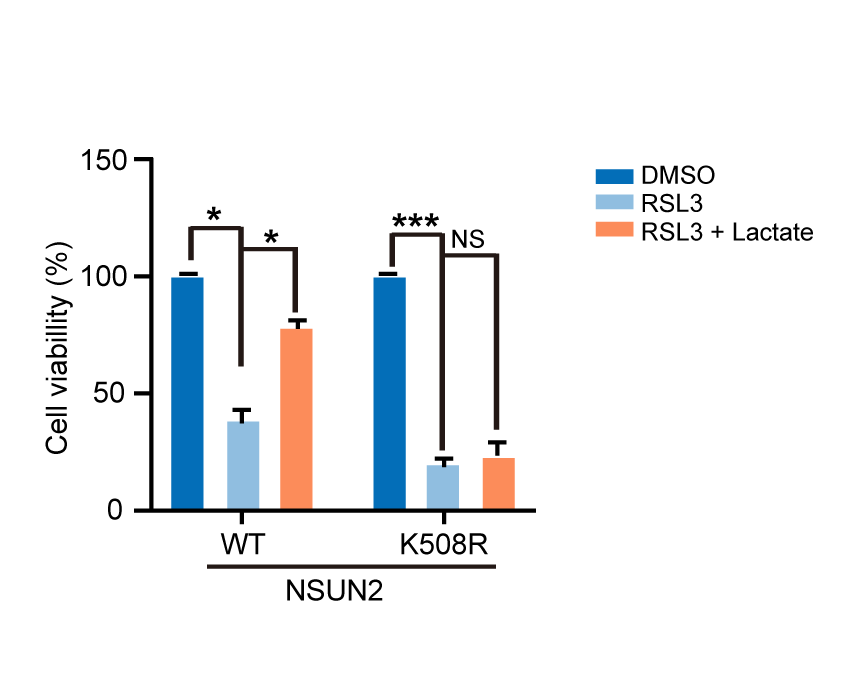


**Figure S6.** Lactate could protect gastric cells from RSL3-induced ferroptosis dependent of NSUN2 lactylation. NSUN2 KO MKN45 cells were transfected with NSUN2 WT or K508R plasmids. Indicated cells were pretreated with 10 mM lactate for 12 h, and then with 20 μM RSL3 for 24 h. The cellular viability were measured by CCK-8. The data represent the mean±SD from three independent experiments. Statistical analysis by two-way ANOVA. **P* < 0.05, ***P* < 0.01, ****P* < 0.001, NS: not significant.


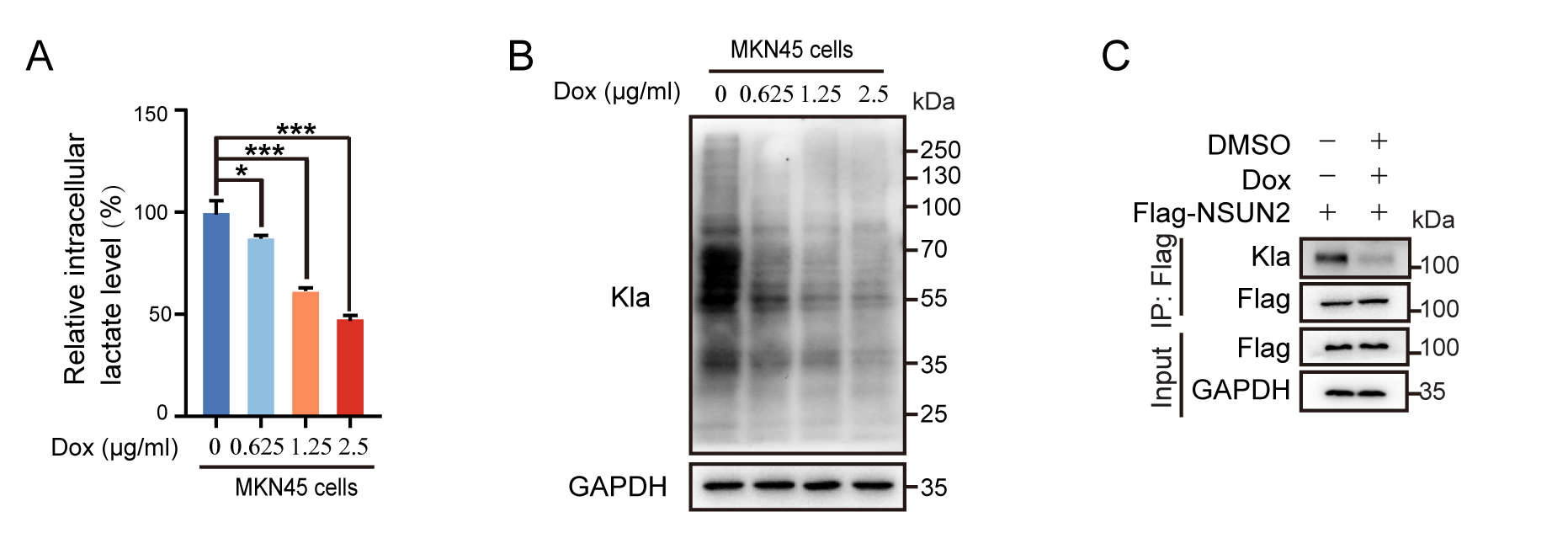


**Figure S7.** The lactylation level of NSUN2 was decreased under Dox treatment. (A and B) The MKN45 cells were treated with Dox in different concentrations (0.625, 1.25, 2.5 μg/ml) for 24 h. The intracellular lactate level (A) and global Kla level (B) were determined. (C) MKN45 cells were transfected with Flag-NSUN2 plasmids and then treated with 1.25 μg/ml Dox. NSUN2 lactylation level was examined in Flag immunoprecipitates with anti-lactyllysine antibody by western blotting. The data represent the mean±SD from three independent experiments. Statistical analysis by two paired Student’s t test. **P* < 0.05, ****P* < 0.001, NS: not significant.


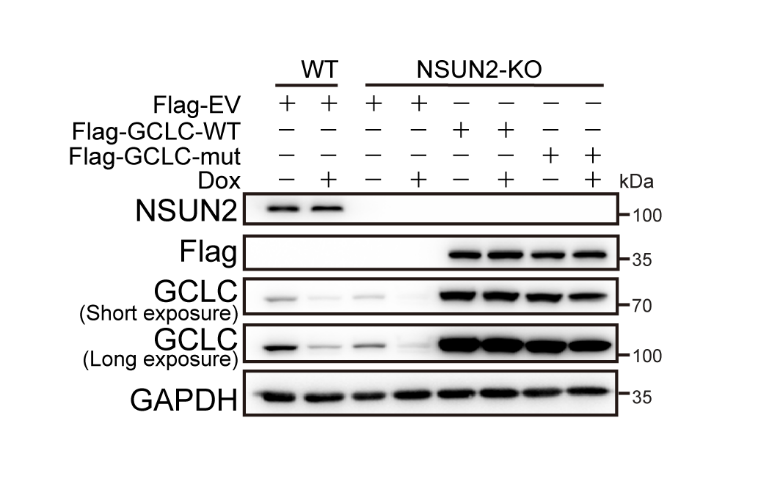


**Figure S8.** The WT or NSUN2 KO cells were transfected with Flag-GCLC-WT or Flag-GCLC-mutation for 36 h. The expression level of indicated proteins validated by western blotting.


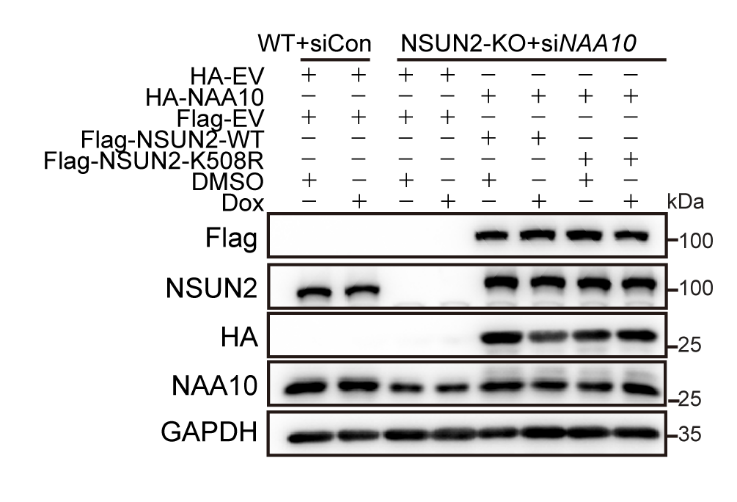


**Figure S9. Overexpression of NSUN2 but not the mutation (K508R) rescues ferroptosis induced by NAA10 and NSUN2 depletion.** The MKN45 NSUN2 KO cells were transfected with siControl or *NAA10* siRNA1 + 2 (si*NAA10*) for 24 h and then transfected with Flag-NSUN2-WT+HA-NAA10 or Flag-NSUN2-K508R + HA-NAA10 for 24 h. The indicated proteins expression levels were validated by western blotting.


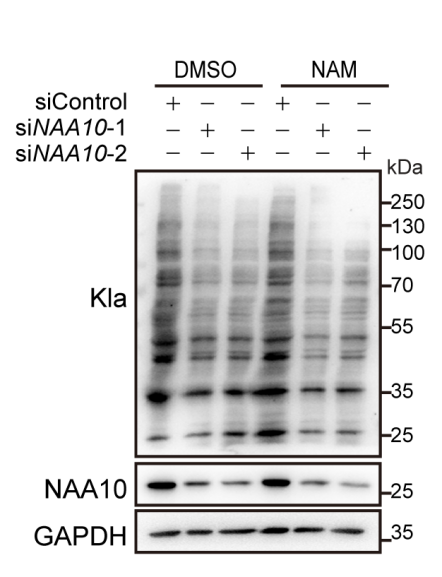


**Figure S10. NAA10 knockdown reduced the global lactylation level of MKN45 cells .** Western blot analysis the total lactylation level of MKN45 cells transfected with siControl or *NAA10* siRNA1 +2 (si*NAA10*) for 48 h, followed by DMSO or 5 μM NAM for 3 h.


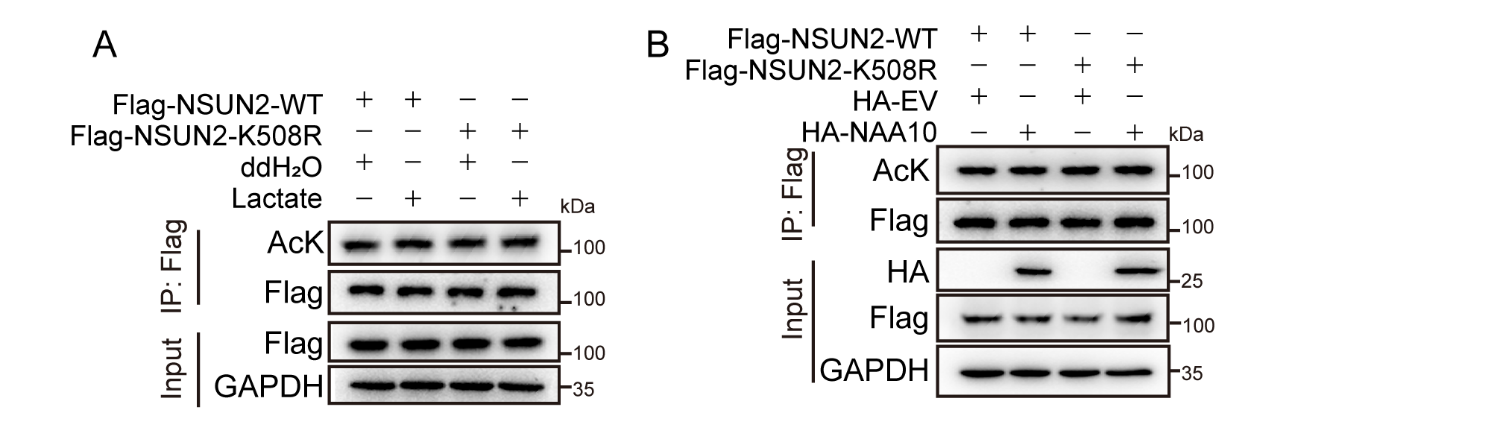


**Figure S11. Lactate treatment did not affect the acetylation level of NSUN2.** (A) HEK293 cells transfected with Flag-NSUN2 WT or mutation K508R were treated with 20 mM lactate for 24 h. Cell lysates were immunoprecipitated using Flag-M2 beads and western blot for acetylation of NSUN2 using anti-acetyllysine antibody. (B) HEK293 cells were transfected with Flag-NSUN2 WT or mutation K508R with HA-EV or HA-NAA10 for 36 h. Cell lysates were immunoprecipitated using Flag-M2 beads and western blot for acetylation of NSUN2 using anti-acetyllysine antibody.


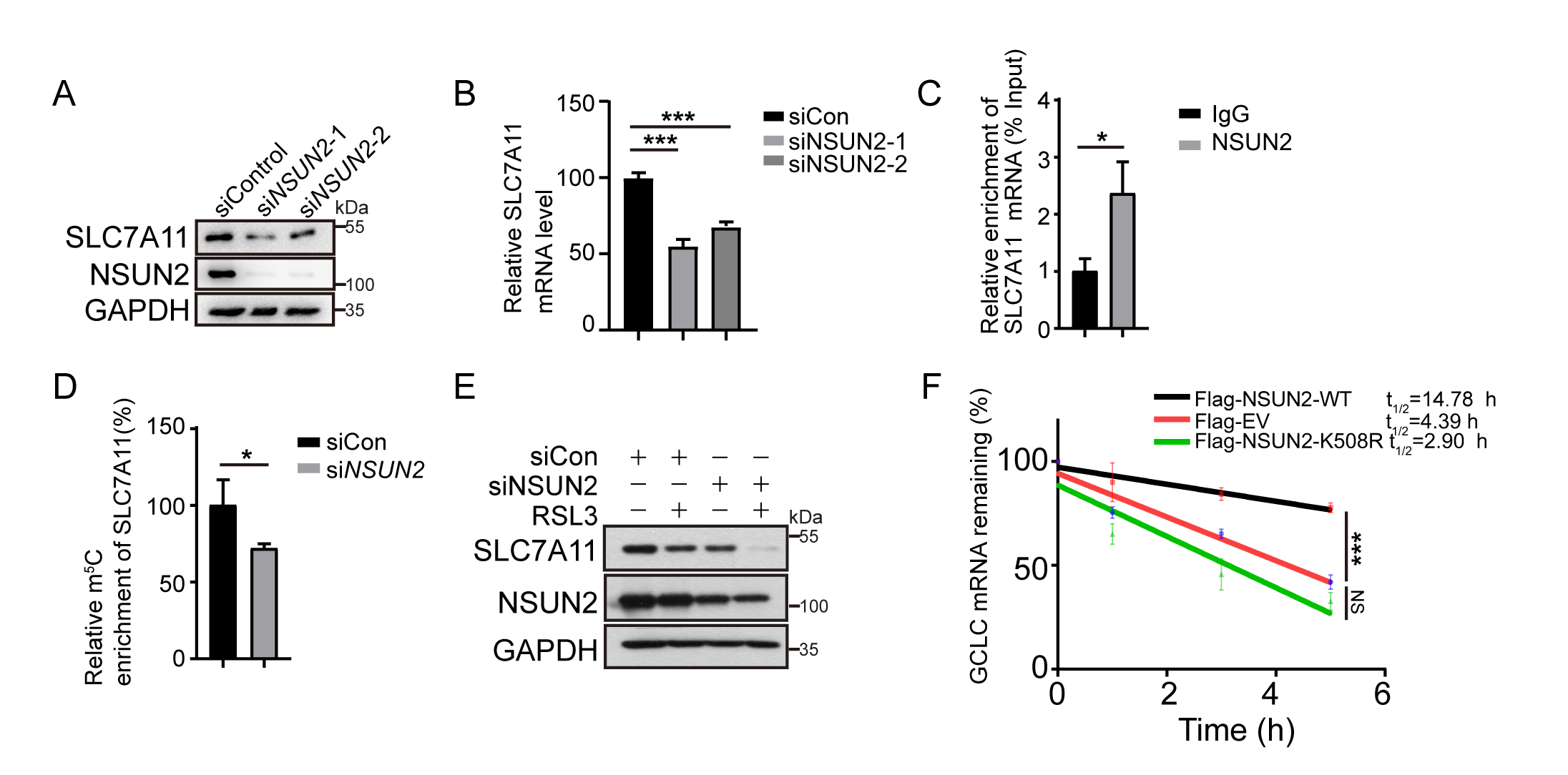


**Figure S12. SLC7A11 was shown to be a target of NSUN2 lactylation during ferroptosis induction. (A)** Western blot analysis of the protein expression level of SLC7A11 after NSUN2 knockdown in MKN45 cells. (B) qRT-PCR analysis that the mRNA expression level of SLC7A11 after NSUN2 knockout in MKN45 cells. The data represent the mean ± SD from three independent experiments. Statistical analysis by paired Student’s t test. ***P < 0.001. (C) RNA immunoprecipitation of SLC7A11 mRNA was carried out in MKN45 cells using anti-NSUN2 antibody, with IgG as the control. The data represent the mean±SD from three independent experiments. Statistical analysis by paired Student’s t test. *P < 0.05. (D) The purified RNA was immunoprecipitated by anti-m^5^C antibody and the m^5^C level of SLC7A11 in MKN45 cells transfected with siControl or siRNA *NSUN2* was analyzed by qRT-PCR. The data represent the mean ± SD from three independent experiments. Statistical analysis by paired Student’s t test. *P < 0.05. (E) MKN45 cells were transfected with siControl or siRNA *NSUN2* for 48 h, and then treated with or without 20 μM RSL3 for 24 h. The expression of SLC7A11 protein was assessed by western blotting. (F) Stability analysis of SLC7A11 mRNA in WT or NSUN2 KO MKN45 cells transfected with Flag-NSUN2-WT or Flag-NSUN2-K508R with treatment of actinomycin D (ActD) for 0, 1, 3 and 6 h. The data represent the mean ± SD from three independent experiments. Statistical analysis by two-way ANOVA. ***P<0.001, NS: not significant.


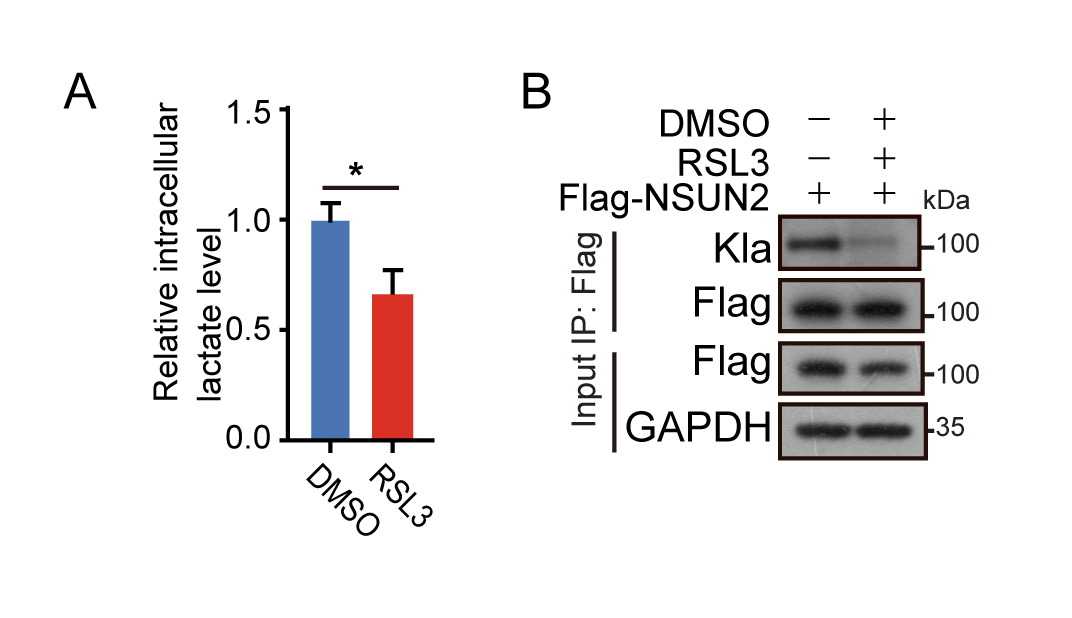


**Figure S13.** The lactylation level of NSUN2 was decreased under RSL3 treatment. The MKN45 cells were transfected with Flag-NSUN2 plasmids for 48 h, and then treated with 20 μM RSL3 for 24 h. (A) Lactate level were measured by L-lactate assay kit; (B) NSUN2 lactylation level was examined in Flag immunoprecipitates with anti-lactyllysine antibody by western blotting. The data represent the mean ± SD from three independent experiments. Statistical analysis by paired Student’s t test. **P* < 0.05.
